# The cancer/testis antigen HORMAD1 mediates epithelial–mesenchymal transition to promote tumor growth and metastasis by activating the Wnt/β-catenin signaling pathway in lung cancer

**DOI:** 10.1038/s41420-022-00946-1

**Published:** 2022-03-28

**Authors:** Kang Liu, Li Cheng, Kun Zhu, Jinhu Wang, Qiang Shu

**Affiliations:** 1 The Children’s Hospital, Zhejiang University School of Medicine, National Clinical Research Center For Child Health, Hangzhou, China; 2 Key Laboratory of Neuropharmacology and Translational Medicine of Zhejiang Province, Zhejiang Chinese Medical University, Hangzhou, China; 3 Department of Pathology, The Children’s Hospital, Zhejiang University School of Medicine, Hangzhou, China; 4 Department of Surgical Oncology, The Children’s Hospital, Zhejiang University School of Medicine, Hangzhou, China; 5 Department of Pediatric Surgery, The Children’s Hospital, Zhejiang University School of Medicine, Hangzhou, China

**Keywords:** Metastasis, Cell signalling

## Abstract

The cancer/testis antigen HORMAD1 is a mechanical regulator that modulates DNA homologous recombination repair and mismatch repair in multiple cancers. However, the role and underlying regulatory mechanisms of HORMAD1 in lung cancer progression remain unknown. Here, we show that HORMAD1 is upregulated in lung adenocarcinoma tissues compared with adjacent normal tissues and that aberrant HORMAD1 expression predicts poor prognosis. We further demonstrate that HORMAD1 promotes the proliferation, migration and invasion of lung cancer cells both in vitro and in vivo by inducing epithelial–mesenchymal transition (EMT). Subsequent mechanistic investigations revealed that HORMAD1 activates the Wnt/β-catenin pathway by increasing the phosphorylation level of AKT at Ser473 and that of GSK-3β at Ser9 in lung cancer cells, which decreases the phosphorylation level of β-catenin at Ser33/37/Thr41, enhances the cytoplasmic and nuclear accumulation of β-catenin and its transcriptional activity, consequently promoting EMT and lung cancer growth and metastasis. Our results provide new insights into the functional role and regulatory mechanism of HORMAD1 in lung cancer progression and identify HORMAD1 as a promising prognostic biomarker and therapeutic target for lung cancer.

## Introduction

Lung cancer is one of the most malignant tumors with increasing incidence and mortality [1, 2]. Although molecular targeted therapy and immunotherapy have allowed revolutionary changes to lung cancer treatment [3], the overall 5-year survival rate is still less than 15% due to its metastatic characteristics [4]. Therefore, identification of the molecular regulators and mechanisms of lung cancer tumorigenesis and progression would also identify novel therapeutic targets for lung cancer.

Accumulating studies have demonstrated that epithelial–mesenchymal transition (EMT) is implicated in various stages of tumor progression, including tumor initiation, invasion, and metastasis [5]. EMT is a biological process in which polarized epithelial cells transform into mesenchymal cells, which enhances the migratory and invasive capabilities of tumor cells [6]. During EMT, the expression of epithelial markers including E-cadherin, tight junction proteins, and cytokeratins, decreases, whereas that of mesenchymal markers, such as N-cadherin, Vimentin, and ZEB-1, increases [7]. The EMT process endows cancer cells with an invasive and migratory mesenchymal phenotype that enables them to enter into the bloodstream and disseminate systemically to other organs [8]. Signaling pathways such as the TGF-β, NF-kB, Wnt/β-catenin, Notch pathways can promote EMT process and tumor metastasis [9]. The canonical Wnt/β-catenin signaling pathway plays a vital role in regulating EMT and lung cancer metastasis [10, 11]. In the absence of Wnt ligands, β-catenin is phosphorylated by the destruction complex containing Axis inhibition protein-2 (Axin-2), APC and glycogen synthase kinase-3β (GSK-3β) and is then ubiquitinated by β-TrCP, culminating in its proteasomal degradation [12]. The Wnt/β-catenin pathway is activated upon binding of Wnt ligands to Fzd receptors and LRP coreceptors, which in turn induces the stabilization and accumulation of cytoplasmic β-catenin followed by its translocation to the nucleus [13]. Following its translocation to the nucleus, β-catenin can interact with T-cell factor/lymphoid enhancer-binding factor-1 to activate the transcription of Wnt target genes, which promotes EMT induction and tumor metastasis [14]. Revealing molecular regulators that regulate the Wnt/β-catenin pathway in lung cancer will potentially benefit the development of therapeutic targets for lung cancer.

HORMAD1, also known as CTA46, is a member of the cancer/testis antigen (CTA) family, whose expression is restricted to germ cells but is aberrant in diverse cancers [15]. CTAs are potential biomarkers and therapeutic targets due to their expression patterns [16]. Studies have revealed that CTAs can promote tumor invasion and metastasis in several cancers [17, 18], but the mechanisms underlying CTA-mediated initiation and progression of cancer remain largely unknown. HORMAD1 is aberrantly expressed in multiple cancers, such as ovarian cancer, breast cancer, and lung cancer. Aberrant HORMAD1 expression in cancer cells has been shown to be associated with genomic instability and DNA damage repair pathway regulation [19–21]. Emerging evidence has demonstrated that HORMAD1 can promote either chemoresistance or chemosensitivity by targeting the homologous recombination repair pathway in different types of cancer [22–24]. Moreover, HORMAD1 has been shown to enhance tumor growth in xenograft models of basal-like breast cancer [25]. However, the precise role of HORMAD1 in lung cancer growth and progression and the potential underlying mechanism has not been investigated.

In this study, we report that HORMAD1 is significantly upregulated in lung cancer tissues compared with the paired noncancerous tissues and that HORMAD1 overexpression predicts poor prognosis in patients. Moreover, we reveal that HORMAD1 promotes the proliferation, migration, and invasion of lung cancer cells both in vitro and vivo. Aberrant HORMAD1 expression stimulates the metastatic properties of lung cancer cells by promoting EMT. In addition, we demonstrate that HORMAD1 activates the Wnt/β-catenin pathway to induce EMT during lung cancer metastasis by regulating AKT/GSK-3β signaling. Furthermore, the Wnt inhibitor XAV939 impairs HORMAD1 overexpression-induced lung cancer cell motility and EMT, and this effect can be reversed by the Wnt activator CHIR99021 in HORMAD1 knockout (KO) cells. Taken together, our study reveals that HORMAD1 functions as a novel regulator of the Wnt/β-catenin signaling pathway in lung cancer progression.

## Results

### HORMAD1 is upregulated in lung cancer and its overexpression predicts poor prognosis of patients

**Fig. 1:**
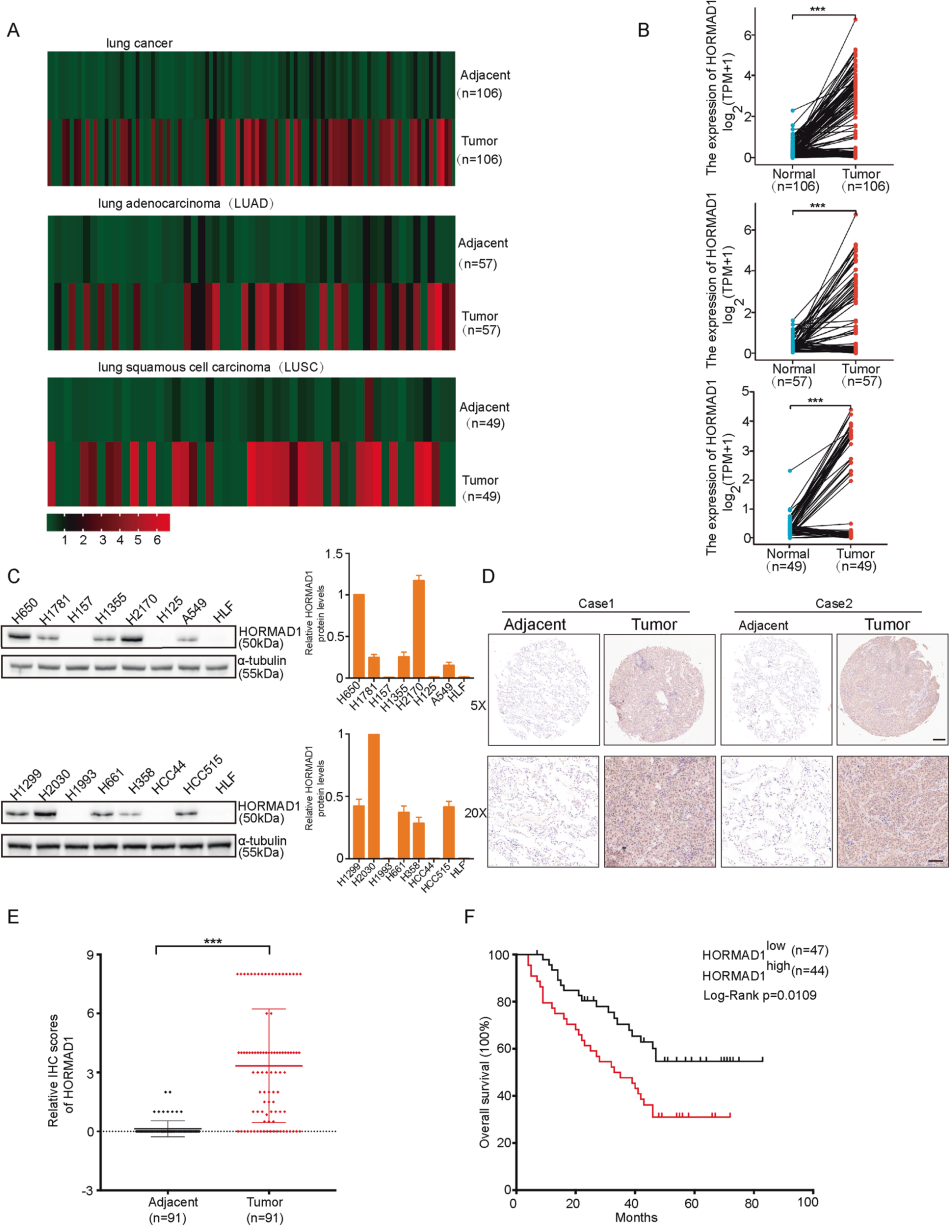
HORMAD1 is upregulated in lung cancer and its overexpression predicts poor prognosis of patients. Bioinformatic analyses of the HORMAD1 expression in pan-lung cancer (*n* = 106), lung adenocarcinoma (*n* = 57), lung squamous cell carcinoma (*n* = 49), and the corresponding normal adjacent nontumor tissues from TCGA datasets, presented on heatmaps (**A**) and scatter plots (**B**). **C** Western blotting analyses of HORMAD1 expression in lung cancer cell lines and the normal human lung fibroblast cell line (HLF). Histograms representing indicated the results of three independent experiments. **D** Representative immunohistochemical (IHC) staining images of HORMAD1 expression in lung adenocarcinoma tissues (*n* = 91) and adjacent normal tissues (*n* = 91) in a lung adenocarcinoma microarray. The scale bars in the 5× magnification and 20× magnification images represent 200 μm and 100 μm, respectively. **E** Graph showing the HORMAD1 staining scores in the range of 0–9 in lung adenocarcinoma tissues and adjacent normal tissues. **F** Kaplan-Meier analysis of the correlation between the HORMAD1 expression level and the overall survival of lung adenocarcinoma patients represented in the tissue array. The patients were stratified by HORMAD1 expression (*P* = 0.0109, log-rank test; *P* < 0.05 was considered significant) into high (IHC score > 4, *n* = 44) and low (IHC score < 4, *n* = 47) expression groups. ***p < 0.001.

To assess the clinical significance of HORMAD1 expression in lung cancer, we first analyzed the HORMAD1 expression level in pan-lung cancer, lung adenocarcinoma and lung squamous cell carcinoma using RNA sequencing (RNA-seq) data from The Cancer Genome Atlas (TCGA). HORMAD1 was significantly upregulated in pan-lung cancer, lung adenocarcinoma, and lung squamous cell carcinoma tissues compared to the paired noncancerous tissues (Fig. 1A, B). We further examined HOMRAD1 expression in various lung cancer cell lines to ascertain its role and found that 10 of the 14 lung cancer cell lines had high HOMRAD1 expression compared with that in the normal human lung fibroblast line (Fig. 1C). To further validate the protein expression of HORMAD1 in lung cancer at different histologic stages, we detected HORMAD1 in a lung adenocarcinoma microarray containing 91 lung adenocarcinoma tissues and corresponding normal lung tissues by immunohistochemistry. HORMAD1 was localized primarily in the cytoplasm and nucleus. Consistent with the above finding, HORMAD1 protein expression in tumor tissues was significantly elevated compared with that in the paired noncancerous tissues (Fig. 1D, E). Kaplan–Meier analysis revealed that high HORMAD1 expression level was associated with poor prognosis of the patients in the lung adenocarcinoma microarray (Fig. 1F). These results demonstrate that HORMAD1 is frequently upregulated in lung cancer and may function as a tumor-promoting factor in human lung cancer.

### HORMAD1 promotes the proliferation, migration, and invasion of lung cancer cells in vitro

**Fig. 2:**
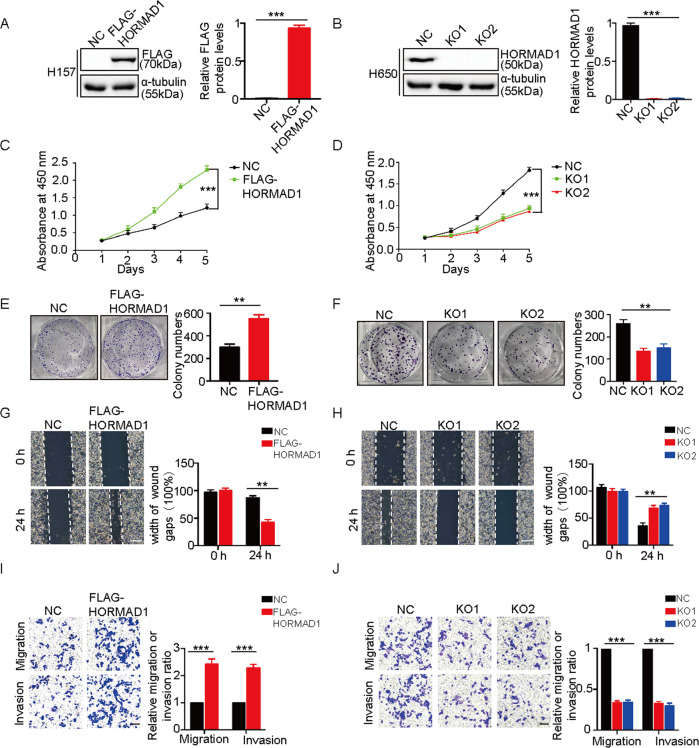
HORMAD1 promotes the proliferation, migration, and invasion of lung cancer cells in vitro. **A** Western blotting and quantitative analyses of HORMAD1 expression in H157 cells with or without HORMAD1 expression. α-tubulin was used as the loading control. **B** Western blotting and quantitative analyses of HORMAD1 expression in HORMAD1 negative control (NC) and KO H650 cells. α-tubulin was used as the loading control. The proliferation of H157 cells with or without HORMAD1 expression (**C**) and NC and HORMAD1 KO H650 cells (**D**) was measured by CCK-8 assays at the indicated time points. The colony-forming ability of H157 cells with or without HORMAD1 expression (**E**) and NC and HORMAD1 KO H650 cells (**F**) was measured by colony formation assays. The migration of H157 cells with or without HORMAD1 expression (**G**) and NC and HORMAD1 KO H650 cells (**H**) was evaluated by wound healing assays; the scale bars are 200 μm. The cell migration and invasion of H157 cells with or without HORMAD1 expression (**I**) and NC and HORMAD1 KO H650 cells (**J**) was examined by Transwell assays; the scale bars are 100 μm. All histograms representing indicated the results of three independent experiments. Mean ± SEM from three independent experiments are shown. **p < 0.01; ***p < 0.001.

To investigate the tumorigenic potential of HORMAD1 in lung cancer cells, we stably overexpressed HORMAD1 in HORMAD1-negative H157 cells and knocked out HORMAD1 in HORMAD1-positive H650 cells using two independent guide RNAs (Fig. 2A, B). The Cell Counting Kit-8 assay demonstrated that HORMAD1 overexpression significantly promoted the growth of HORMAD1-negative H157 lung cancer cells, whereas HORMAD1 KO markedly compromised the growth of HORMAD1-expressing H650 lung cancer cells (Fig. 2C, D). Similarly, the results of the colony formation assay revealed that HORMAD1 overexpression significantly increased the colony-forming capacity of H157 cells, while HORMAD1 KO markedly decreased the colony-forming capacity of H650 cells (Fig. 2E, F). Consistently, overexpression of HORMAD1 increased the growth and colony-forming capacity in HORMAD1-negatived H125 cells while these effects were decreased when HORMAD1 expression was knocked down in HORMAD1-expressing H2170 cells (Fig. S1A–F). These results demonstrate that HORMAD1 promotes the proliferation of lung cancer cells in vitro.

To determine whether HORMAD1 affects the migration and invasion of lung cancer cells, wound healing and Transwell assays were performed. The migration and invasion abilities of H157 lung cancer cells were significantly increased after HORMAD1 overexpression, and those of H650 cells were significantly decreased after HORMAD1 KO (Fig. 2G–J). Collectively, these data suggest that HORMAD1 promotes the migration and invasion of lung cancer cells in vitro.

### HORMAD1 promotes tumor growth and metastasis in vivo

**Fig. 3:**
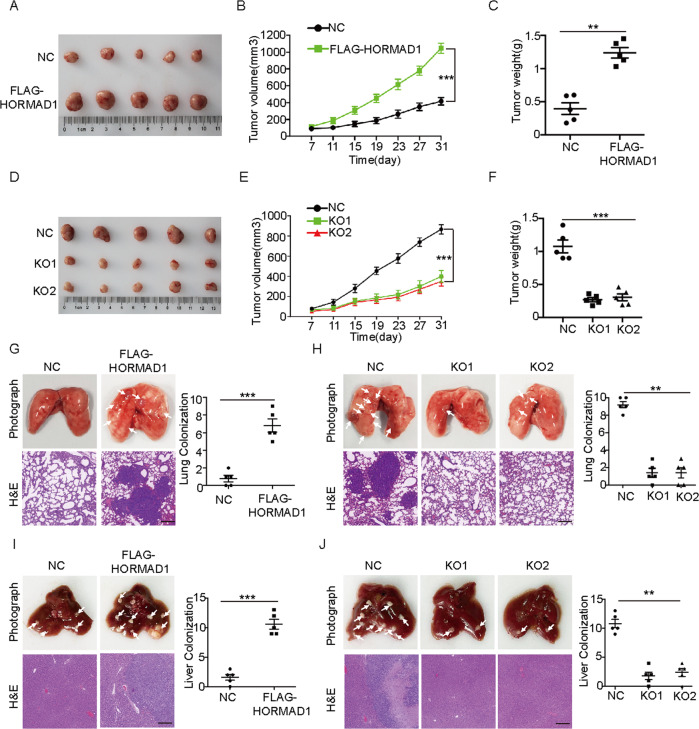
HORMAD1 promotes tumor growth and metastasis in vivo. **A** Photographs of xenografts tumors from each nude mouse in the H157-HORMAD1 group and vector control group. *n* = 5. **B** Tumor growth curves for the H157-HORMAD1 group and vector control group at the indicated times. *n* = 5. **C** Tumor weights in the H157-HORMAD1 group and vector control group. *n* = 5. **D** Photographs of xenografts tumors from each nude mouse in the H650-NC and H650-HORMAD1 KO groups. *n* = 5. **E** Tumor growth curves for the H650-NC and H650-HORMAD1 KO groups at the indicated times. *n* = 5. **F** Tumor weights in the H650-NC and H650-HORMAD1 KO groups. *n* = 5. Representative gross appearance of the lungs (upper) in the H157-HORMAD1 group and vector control groups (**G**) and the H650-NC and H650-HORMAD1 KO groups (**H**); H&E staining of lung metastases (lower) and quantification of lung metastatic nodules (right). The metastatic nodules are indicated by arrows and scale bars are 200 μm. *n* = 5. Representative gross appearance of the livers (upper) in the H157-HORMAD1 group and vector control groups (**I**) and the H650-NC and H650-HORMAD1 KO groups (**J**); H&E staining of liver metastases (lower) and quantification of liver metastatic nodules (right). The metastatic nodules are indicated by arrows and scale bars are 200 μm. *n* = 5. Mean ± SEM from three independent experiments are shown. **p < 0.01; ***p < 0.001.

To explore the protumorigenic role of HORMAD1 in vivo, we subcutaneously injected the H157-HORMAD1 and H650-HORMAD1 KO cells and the corresponding control cells into the armpit of BALB/c-nude mice to evaluate the effect of HORMAD1 on tumor growth. The results showed that mice injected with H157-HORMAD1 cells exhibited significantly greater tumor weight and volume than mice injected with vector control cells (Fig. 3A–C). In contrast, the tumor weight and volume were significantly decreased in mice injected with H650-HORMAD1 KO cells compared to mice injected with vector control cells (Fig. 3D–F). These results suggest that HORMAD1 promotes tumor growth in vivo.

To explore whether HORMAD1 promotes tumor metastasis in vivo, we injected the indicated cells into the tail vein or the subcapsular region of the nude mice to develop lung and liver metastasis models [26], respectively. The numbers of lung and liver metastatic nodules were significantly increased in mice injected with the H157-HORMAD1 cells compared with those injected with vector control cells, whereas fewer lung and liver metastatic nodules were formed in mice injected with H650-HORMAD1 KO cells than in mice injected with vector control cells (Fig. 3G–J). These results suggested that HORMAD1 promotes the invasion and metastasis of lung cancer cells in vivo.

### HORMAD1 promotes EMT in lung cancer cells

**Fig. 4:**
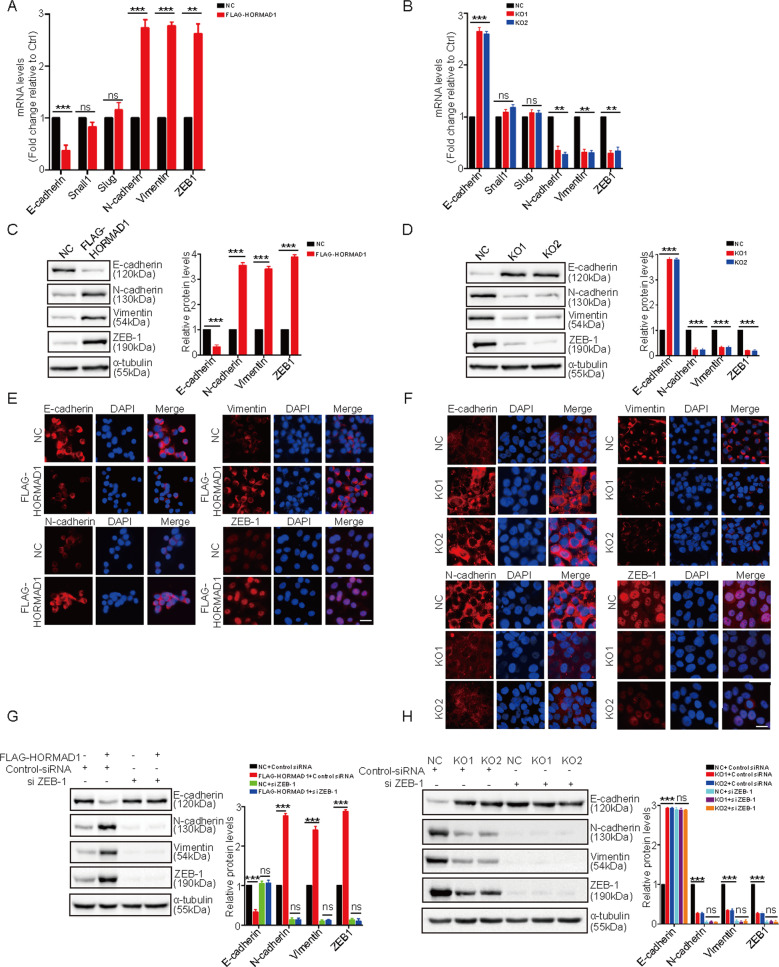
HORMAD1 promotes epithelial–mesenchymal transition (EMT) in lung cancer cells. The relative mRNA levels of E-cadherin, Snail1, Slug, N-cadherin, Vimentin, and ZEB-1 in H157 cells with or without HORMAD1 expression (**A**) and in NC and HORMAD1 KO H650 cells (**B**) were determined by quantitative real-time PCR. The protein levels of E-cadherin, N-cadherin, Vimentin, and ZEB-1 in H157 cells with or without HORMAD1 expression (**C**) and in NC and HORMAD1 KO H650 cells (**D**) were analyzed by western blotting. Immunofluorescence staining analyses of E-cadherin, N-cadherin, Vimentin, and ZEB-1 in H157 cells with or without HORMAD1 expression (**E**) and in NC and HORMAD1 KO H650 cells (**F**); scale bars are 100 μm. The protein levels of E-cadherin, N-cadherin, Vimentin, and ZEB-1 in H157 cells with or without HORMAD1 expression (**G**) and in NC and HORMAD1 KO H650 cells (**H**) were analyzed by western blotting after transfection with ZEB-1 siRNA. All histograms representing indicated the results of three independent experiments. Mean ± SEM from three independent experiments are shown. **p < 0.01; ***p < 0.001; ns, not significant.

Previous studies have shown that cancer/testis antigens induce EMT, which is associated with cancer progression and metastasis [27, 28]. We then investigated whether HORMAD1 promotes lung cancer cell migration and invasion by regulating EMT. We examined the mRNA expression levels of an epithelial marker (E-cadherin) and mesenchymal markers (N-cadherin, Vimentin, Slug, Snail, and ZEB-1) by quantitative real-time PCR. Overexpression of HORMAD1 in H157 cells decreased the mRNA expression level of E-cadherin and increased those of N-cadherin, Vimentin, and ZEB-1 (Fig. 4A). Conversely, knockout of HORMAD1 in H650 cells showed the opposite effects (Fig. 4B). Notably, the mRNA expression levels of Snail1 and Slug were not affected by HORMAD1 expression (Fig. 4A, B). The western blotting results confirmed that HORMAD1 reduced the protein expression level of E-cadherin and increased those of N-cadherin, Vimentin, and ZEB-1 (Figs. 4C, D, S2A, B). Consistently, the immunofluorescence staining results indicated that HORMAD1 promoted a significant increase in the fluorescence intensity of N-cadherin, Vimentin, and ZEB-1 as well as a decrease in the fluorescence intensity of E-cadherin (Fig. 4E, F). These observations suggest that HORMAD1 induces EMT in lung cancer cells.

Several transcription factors, including Snail1, Slug, and ZEB-1, are involved in the repression of E-cadherin in cancer cells [29–31]. Given that HORMAD1 increased the expression of ZEB-1, but not Snail1 or Slug, we hypothesized that HORMAD1 promotes EMT in lung cancer cells by increasing the expression of ZEB-1. To test this hypothesis, we knocked down ZEB-1 expression using a specific small interfering RNA (siRNA) in H157-HORMAD1 and H650-HORMAD1 KO cells and the corresponding control cells. The results indicated that knockdown of ZEB-1 reversed the HORMAD1-mediated downregulation of E-cadherin and upregulation of N-cadherin and Vimentin (Figs. 4G-H, S2C, D). Taken together, these data demonstrate that HORMAD1 promotes EMT in a ZEB-1-dependent manner.

### HORMAD1 activates the Wnt/β-catenin pathway in lung cancer cells

**Fig. 5:**
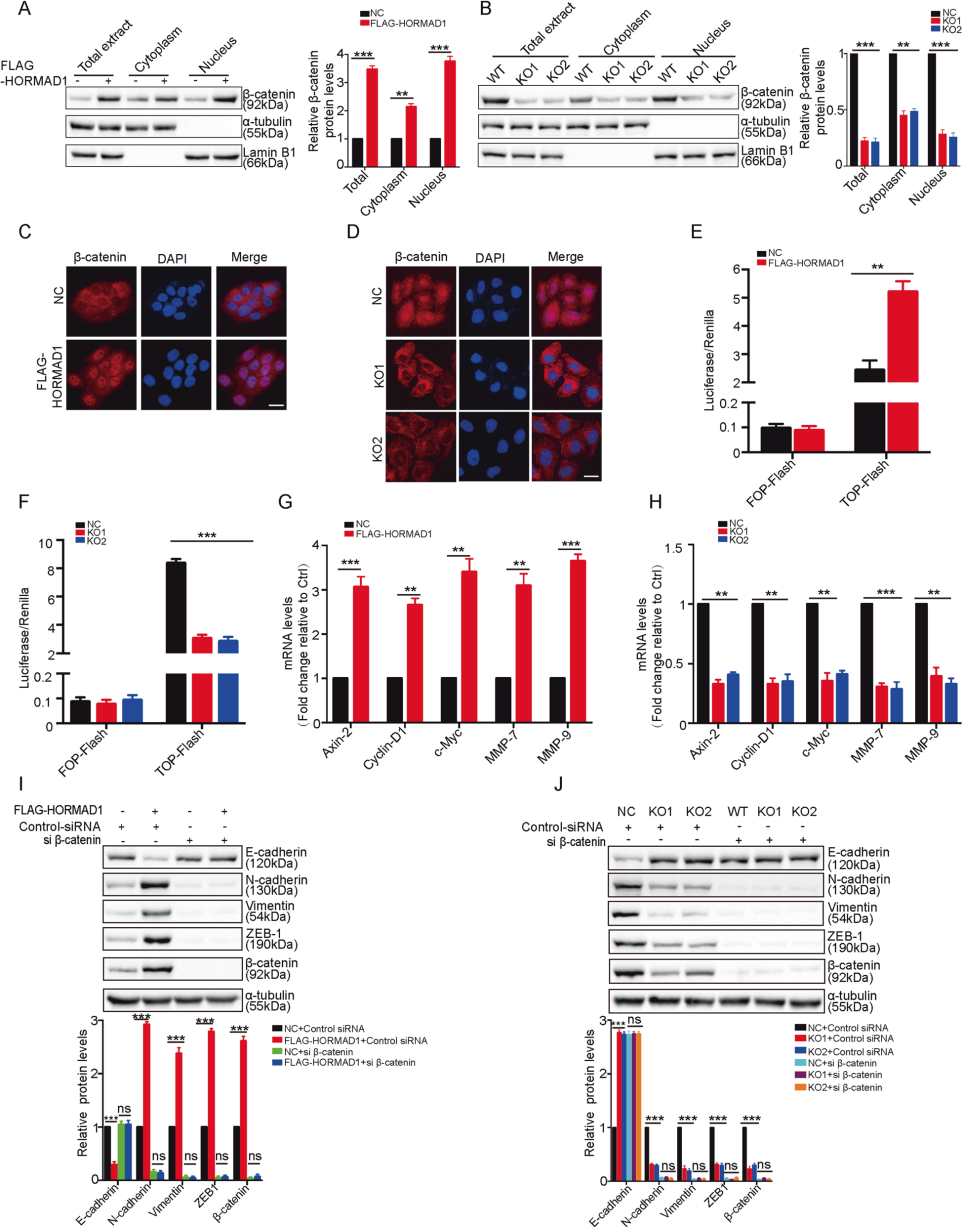
HORMAD1 activates the Wnt/β-catenin pathway in lung cancer cells. Western blotting and quantitative analyses of total, cytosolic, and nuclear β-catenin in H157 cells with or without HORMAD1 expression (**A**) and in NC and HORMAD1 KO H650 cells (**B**). α-tubulin and Lamin B1 were used as the loading controls for the cytoplasmic and nuclear fractions, respectively. Immunofluorescence staining analyses of the subcellular β-catenin localization in H157 cells with or without HORMAD1 expression (**C**) and in NC and HORMAD1 KO H650 cells (**D**); scale bars are 100 μm. TOP-Flash/FOP-Flash luciferase reporter assay of β-catenin transcriptional activity in H157 cells with or without HORMAD1 expression (**E**) and in NC and HORMAD1 KO H650 cells (**F**). The relative mRNA levels of Axin-2, Cyclin-D1, c-Myc, MMP-7, and MMP-9 in H157 cells with or without HORMAD1 (**G**) and NC and HORMAD1 KO H650 cells (**H**) were analyzed by quantitative real-time PCR. Western blotting and quantitative analyses of E-cadherin, N-cadherin, Vimentin, ZEB-1, and β-catenin protein levels in H157 cells with or without HORMAD1 expression (**I**) and in NC and HORMAD1 KO H650 cells (**J**) after transfection with β-catenin siRNA. All histograms representing indicated the results of three independent experiments. Mean ± SEM from three independent experiments are shown. **p < 0.01; *** *p* < 0.001; ns, not significant.

Previous studies have reported that the Wnt/β-catenin pathway is involved in the regulation of EMT induction and tumor metastasis [9, 32]. Our results demonstrated that HORMAD1 promotes EMT by increasing the expression of ZEB-1. Since recent studies suggested that β-catenin is an upstream regulator of ZEB-1 [30, 33], we hypothesized that HORMAD1 promotes ZEB1-mediated EMT through regulation of the Wnt/β-catenin pathway. Given that regulation of β-catenin protein level is the key feature of Wnt/β-catenin pathway, we sought to determine whether HORMAD1 affects the β-catenin protein level in lung cancer cells. HORMAD1 overexpression in H157 cells increased but HORMAD1 KO in H650 cells reduced the total β-catenin level. We then separated the cytosolic and nuclear fractions from H157-HORMAD1 and H650-HORMAD1 KO cells and the corresponding control cells and found that the amounts of β-catenin in the cytoplasmic and nucleus were significantly increased in H157 and H125 cells after HORMAD1 overexpression. Conversely, the amounts of β-catenin in the cytosol and nucleus were significantly decreased in H650 and H2170 cells after HORMAD1 KO (Figs. 5A, B, S3A, B). Moreover, immunofluorescence assay was performed to detect the subcelluar localization of β-catenin. The results indicated that HORMAD1 overexpression in H157 cells significantly increased the nuclear localization of β-catenin. However, the nuclear localization of β-catenin was significantly decreased after HORMAD1 KO in H650 cells (Fig. 5C, D). We then performed TOP-Flash and FOP-Flash luciferase reporter assays to investigate the transcriptional activity of β-catenin. HORMAD1 overexpression significantly increased but HORMAD1 KO significantly decreased the transcriptional activity of β-catenin (Fig. 5E, F). Next, the mRNA levels of Wnt/β-catenin signaling targets, including Axin2, Cyclin-D1, c-Myc, MMP-7, and MMP-9, were measured by quantitative real-time PCR. As shown in Fig. 5G–H, overexpression of HORMAD1 increased but KO of HORMAD1 reduced the mRNA levels of these target genes.

To determine whether HORMAD1-induced EMT depends on β-catenin, we knocked down β-catenin in H157-HORMAD1 and H650-HORMAD1 KO cells and the corresponding control cells using a specific siRNA. Knockdown of β-catenin reversed the HORMAD1-mediated downregulation of E-cadherin and upregulation of N-cadherin, Vimentin, and ZEB-1, suggesting that HORMAD1 overexpression induces EMT through β-catenin (Figs. 5I-J, S3C, D). Collectively, these results demonstrate that HORMAD1 promotes the expression of β-catenin by increasing its accumulation in the cytoplasm and nucleus, thereby leading to activation of the Wnt/β-catenin signaling pathway and induction of EMT.

### HORMAD1 activates the Wnt/β-catenin pathway by regulating AKT/GSK-3β signaling

**Fig. 6:**
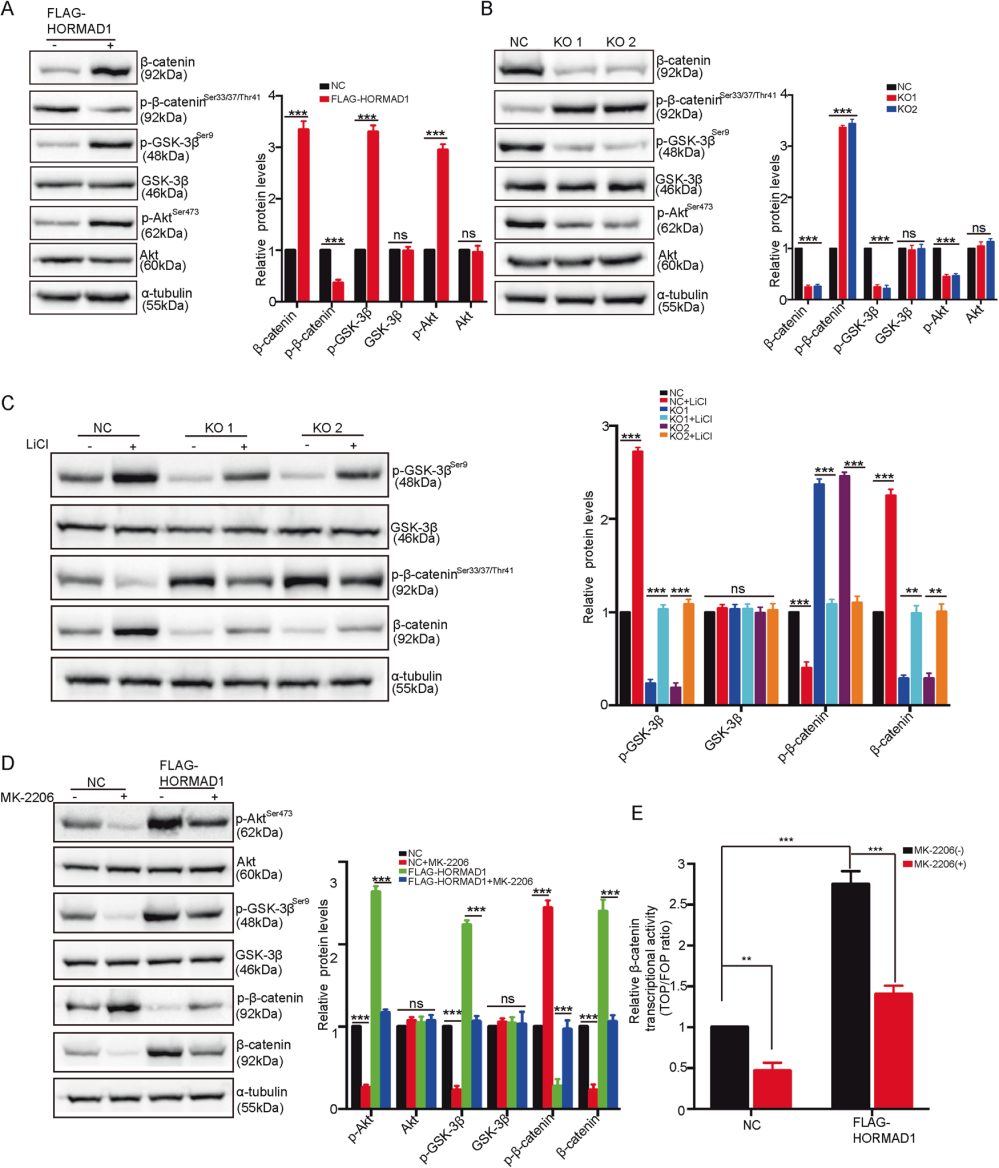
HORMAD1 activates the Wnt/β-catenin pathway by regulating AKT/GSK-3β signaling. Western blotting and quantitative analyses of β-catenin, p-β-catenin^Ser33/37/Thr41^, p-GSK-3β^Ser9^, GSK-3β, AKT, and p-AKT^ser473^ protein levels in H157 cells with or without HORMAD1 expression (**A**) and in NC and HORMAD1 KO H650 cells (**B**). α-tubulin was used as the loading control. **C** Western blotting and quantitative analyses of p-GSK-3β^Ser9^, GSK-3β, p-β-catenin^Ser33/37/Thr41^, and β-catenin protein levels in NC and HORMAD1 KO H650 cells after 24 h of treatment with 20 mM LiCl. α-tubulin was used as the loading control. **D** Western blotting and quantitative analyses of p-AKT^ser473^, AKT, p-GSK-3β^Ser9^, GSK-3β, p-β-catenin^Ser33/37 Thr41^, and β-catenin protein levels in H157 cells with or without HORMAD1 expression after 24 h of treatment with 10 μM MK-2206. α-tubulin was used as the loading control. **E**.TOP-Flash/FOP-Flash luciferase reporter assay of β-catenin transcriptional activity in H157 cells with or without HORMAD1 expression after 24 h of treatment with 10 μM MK-2206. All histograms representing indicated the results of three independent experiments. Mean ± SEM from three independent experiments are shown. **p < 0.01; ***p < 0.001; ns, not significant.

The above results strongly suggest that HORMAD1 activates the Wnt/β-catenin pathway by increasing the amounts of β-catenin in the cytoplasm and nucleus and enhancing its transcriptional activity, but the underlying mechanisms require further investigation. Since phosphorylation of β-catenin at Ser33/37/Thr41 by GSK-3β is necessary for its degradation and Wnt/β-catenin signaling activation [12, 34], we examined the phosphorylation status of β-catenin. Overexpression of HORMAD1 in H157 cells significantly decreased but KO of HORMAD1 in H650 cells significantly increased the phosphorylation of β-catenin at Ser33/37/Thr41 (Fig. 6A, B). Previous studies suggested that AKT phosphorylates GSK-3β at Ser9 and then deactivates it, which in turn increases the cytoplasmic and nuclear localization of β-catenin and the transcriptional activity of Wnt/β-catenin signaling [35, 36]. To determine whether HORMAD1 activates the Wnt/β-catenin pathway through AKT/GSK-3β signaling, we then detected the expression and phosphorylation status of AKT and GSK-3β. The levels of total AKT and GSK-3β were not affected by HORMAD1 expression. Interestingly, HORMAD1 overexpression in H157 cells significantly increased but HORMAD1 KO in H650 cells significantly decreased the phosphorylation of AKT at Ser473 and GSK-3β at Ser9 (Fig. 6A, B). Furthermore, the effects of HORMAD1 KO on decreasing the expression levels of p-GSK-3β (Ser9) and β-catenin and increasing the expression level of p-β-catenin (Ser33/37/Thr41) were partially reversed by treatment with the GSK-3β inhibitor Licl, suggesting that HORMAD1 decreases the phosphorylation and degradation of β-catenin in a GSK-3β-dependent manner (Fig. 6C). In addition, treatment of H157-HORMAD1 cells with the AKT inhibitor MK-2206 reduced AKT and GSK-3β phosphorylation to some extent, thus partially rescuing β-catenin phosphorylation and the total β-catenin level (Fig. 6D). Similarly, the elevated transcriptional activity of β-catenin induced by HORMAD1 overexpression was significantly repressed by MK-2206, suggesting that the HORMAD1 overexpression-induced increase in the transcriptional activity of β-catenin depends on AKT/GSK-3β signaling (Fig. 6E). Taken together, these results demonstrate that HORMAD1 promotes the phosphorylation of AKT at Ser473 and GSK-3β at Ser9, which decreases β-catenin phosphorylation and increases the expression of β-catenin in the cytoplasm and nucleus, leading to transcriptional activation of the Wnt/β-catenin pathway.

### HORMAD1 promotes EMT and metastasis through the Wnt/β-catenin pathway

**Fig. 7:**
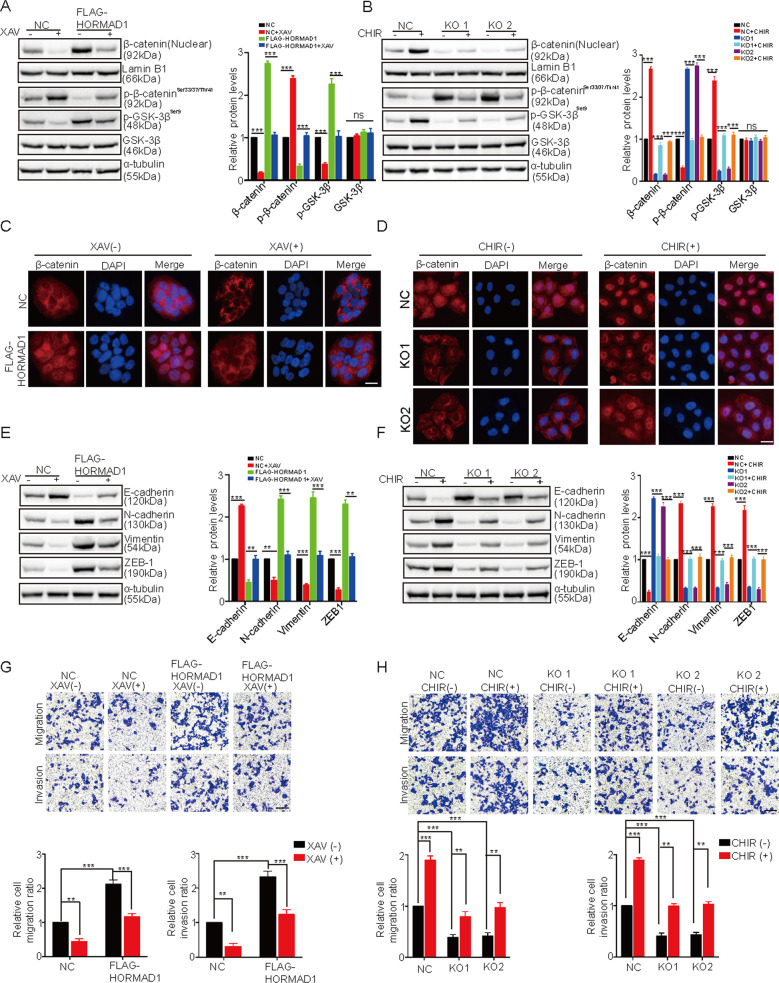
HORMAD1 promotes EMT and metastasis through the Wnt/β-catenin pathway. **A** Western blotting and quantitative analyses of nuclear β-catenin, p-β-catenin^Ser33/37/Thr41^, p-GSK-3β^Ser9^, and GSK-3β protein levels in H157 cells with or without HORMAD1 expression after 24 h of treatment with 15 μM XAV939. α-tubulin and Lamin B1 were used as the loading controls. **B** Western blotting and quantitative analyses of nuclear β-catenin, p-β-catenin^Ser33/37/Thr41^, p-GSK-3β^Ser9^, and GSK-3β protein levels in NC and HORMAD1 KO H650 cells after 24 h of treatment with 6 μM CHIR99021. α-tubulin and Lamin B1 were used as the loading controls. **C** Immunofluorescence staining analyses of the subcellular β-catenin localization in H157 cells with or without HORMAD1 expression after 24 h of treatment with 15 μM XAV939; scale bars are 100 μm. **D** Immunofluorescence staining analyses of subcellular β-catenin localization in NC and HORMAD1 KO H650 cells after 24 h of treatment with 6 μM CHIR99021, scale bars are 100 μm. **E** Western blotting and quantitative analyses of E-cadherin, N-cadherin, Vimentin, and ZEB-1 protein levels in H157 cells with or without HORMAD1 after 24 h of 15 μM XAV-939 treatment. α-tubulin was used as the loading control. **F** Western blotting and quantitative analyses of E-cadherin, N-cadherin, Vimentin, and ZEB-1 protein levels in H650 NC and HORMAD1 KO cells after 24 h of treatment with 6 μM CHIR99021. α-tubulin was used as the loading control. **G**. The migration and invasion of H157 cells with or without HORMAD1 expression were examined after 24 h of treatment with 15 μM XAV939. **H** The migration and invasion of NC and HORMAD1 KO H650 cells were examined after 24 h of treatment with 6 μM CHIR99021. All histograms representing indicated the results of three independent experiments. Mean ± SEM from three independent experiments are shown. **p < 0.01; ***p < 0.001; ns, not significant.

To further investigate whether HORMAD1 promotes EMT and metastatic behaviors in lung cancer cells via the Wnt/β-catenin pathway, we treated H157-HORMAD1 and H650-HORMAD1 KO cells with the Wnt/β-catenin signaling specific inhibitor XAV939 or activator CHIR99021, respectively. XAV939 treatment decreased the level of p-GSK-3β (Ser9) and increased the level of p-β-catenin (Ser33/37/Thr41), while CHIR99021 treatment produced the opposite effects (Fig. 7A, B). Strikingly, XAV939 rescued GSK-3β (Ser9) and β-catenin (Ser33/37/Thr41) phosphorylation in H157-HORMAD1 cells, and CHIR99021 restored GSK-3β (Ser9) and β-catenin (Ser33/37/Thr41) phosphorylation in H650-HORMAD1 KO cells (Fig. 7A, B). We also observed that XAV939 decreased but CHIR99021 increased nuclear β-catenin accumulation. Similarly, XAV939 impaired β-catenin nuclear accumulation in H157-HORMAD1 cells, and CHIR99021 rescued β-catenin nuclear accumulation in H650-HORMAD1 KO cells (Fig. 7C, D). We then evaluated the effects of XAV939 and CHIR99021 on EMT markers. XAV939 decreased the expression of N-cadherin, Vimentin, and ZEB-1 and increased the expression of E-cadherin. CHIR99021 had the opposite effects. Moreover, XAV939 neutralized EMT promotion resulting from HORMAD1 overexpression (Fig. 7E), while CHIR99021 antagonized EMT inhibition caused by HORMAD1 KO (Fig. 7F). In addition, XAV939 reduced the enhancement of migration and invasion induced by HORMAD1 overexpression, and CHIR99021 restored the migration and invasion capacities reduced by HORMAD1 KO (Fig. 7G, H). Collectively, these results demonstrate that HORMAD1 promotes lung cancer cell EMT and motility through the Wnt/β-catenin pathway.

## Discussion

**Fig. 8:**
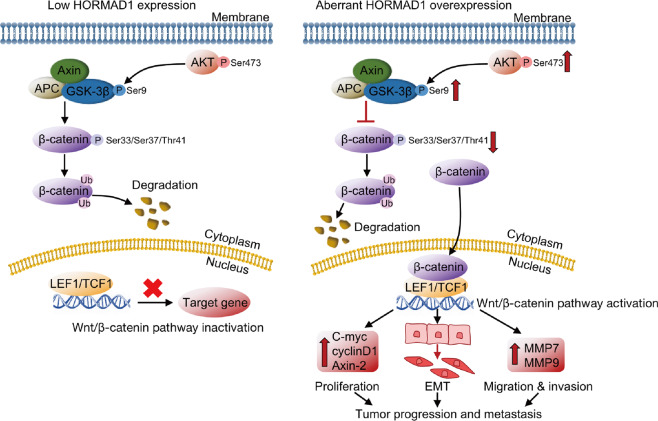
Schematic model of the mechanism by which HORMAD1 functions to promote the malignant phenotypes of lung cancer cells. Aberrant HORMAD1 expression suppresses the degradation of cytosolic β-catenin and promotes nuclear β-catenin accumulation by regulating AKT/GSK-3β signaling to activate the Wnt/β-catenin pathway, accompanied with upregulation of downstream target genes and induction of EMT to promote tumor growth and metastasis in lung cancer.

The role of HORMAD1 in DNA repair and the chemotherapeutic response has been reported in various cancers, including ovarian cancer, basal-like and triple-negative breast cancer, and lung cancer [19–24]. However, the detailed biological functions of HORMAD1 in lung cancer progression and the underlying mechanisms remain unknown. This study is the first to show the function and mechanism of HORMAD1 in lung cancer progression. We revealed a new oncoprotein expression profile of HORMAD1 in lung cancer and demonstrated that HORMAD1 is significantly upregulated in lung cancer, and that its overexpression is associated with poor prognosis. Mechanistically, aberrantly expressed HORMAD1 in lung cancer cells activates the Wnt/β-catenin signaling pathway to promote EMT by regulating of AKT/GSK-3β signaling, which enhances cytoplasmic accumulation and nuclear translocation of β-catenin and increases the expression of the Wnt target genes to promote lung cancer growth and metastasis (Fig. 8).

Our study showed that HORMAD1 expression in lung cancer tissues is evidently increased compared to that in normal tissues and that increased HORMAD1 expression results in unfavorable prognostic outcomes in lung adenocarcinoma patients. A recent study by Zong et al. has showed that HORMAD1 silencing did not affect cell proliferation and prognosis of patients in Triple Negative Breast Cancers [21]. However, our results and the work of Nichols et al. demonstrated that HORMAD1 promotes the proliferation of lung cancer cells and its overexpression predicts poor prognosis of patients [22]. These differences suggested that the function of HORMAD1 might be dependent on cancer type. Landemaine and colleagues predicted previously that HORMAD1 and other genes correlate with lung metastasis in breast cancer [37]. However, no study has yet attempted to investigate functional participation of HORMAD1 in lung cancer metastasis. In this study, our results revealed that HORMAD1 promotes tumor growth and metastasis in vivo using xenograft models and models of liver and lung metastasis. Collectively, our results suggest that aberrantly overexpressed HORMAD1 leads to lung cancer growth and metastasis by acting as a oncogenic regulator.

More than 90% of cancer-associated deaths are caused by metastasis, and EMT has been demonstrated to play critical roles in metastasis [7]. Activation of EMT enables cancer cells to migrate and invade beyond the extracellular matrix of the surrounding tissue in the metastatic cascade [38]. Emerging studies have revealed that numerous CTAs, such as SSX (CTA5), CAGE-1 (CTA3), Piwil2 (CTA80), CT45A1, and the MAGE family gene MAGE-C2 (CTA10), induce EMT to promote tumor metastasis [27, 28]. However, to date, no studies have revealed the role of HORMAD1 as a CTA in EMT and tumor metastasis. Given that HORMAD1 promoted the migration and invasion of lung cancer cells, we hypothesized that HORMAD1 might stimulate lung cancer metastasis by regulating EMT. Our results herein demonstrated that overexpression of HORMAD1 significantly increased the expression of ZEB-1, N-cadherin, and Vimentin and decreased the expression of E-cadherin in lung cancer cells. The opposite effects were observed in HORMAD1 KO lung cancer cells. Moreover, HORMAD1-mediated regulation of EMT-related markers was eliminated by ZEB-1 knockdown, suggesting that ZEB1 is required for HORMAD1-induced EMT process in lung cancer cells. For the first time, our study revealed that aberrantly expressed HORMAD1 may function as an EMT inducer to promote lung cancer metastasis.

Aberrant activation of Wnt/β-catenin signaling promotes EMT in various cancers [39]. In lung cancer, unlike in other cancer types, mutations in β-catenin and other components of the Wnt/β-catenin pathway are not frequently detected, and mutations in β-catenin can be detected only 2% of lung adenocarcinomas [40]. However, activation of the Wnt/β-catenin signaling pathway was widely observed in lung cancer cells and tissues, indicating that other modulators regulating Wnt/β-catenin signaling are prevalent in lung cancer [41, 42]. Thus, identifying and understanding the specific modulators that regulate the Wnt/β-catenin pathway in lung cancer cells may provide new insights into the molecular mechanisms underlying EMT and tumor metastasis, and may lead to the development of novel therapeutic targets for lung cancer. This study revealed that HORMAD1 promotes the expression of β-catenin by increasing its cytoplasmic accumulation and subsequent nuclear translocation, leading to activation of Wnt/β-catenin signaling. Our results suggested that HORMAD1 is a novel regulator upstream of Wnt/β-catenin signaling. Given that β-catenin has been generally defined as an undruggable target because it has large flat surfaces and lacks deep binding pockets for small molecule binding, identification of the upstream factors of β-catenin may provide potential drug targets [43, 44]. However, the potential of HORMAD1 as a therapeutic target requires further evaluation. Aberrant Wnt/β-catenin signaling is involved in the progression of various cancer types via regulation of the downstream Wnt-responsive genes. Our results demonstrated that HORMAD1 expression increased the expression of Axin-2, Cyclin-D1, c-Myc, MMP-7, and MMP-9. Among these target genes, c-myc and Cyclin D1 are involved in cell proliferation [45, 46] and MMP-7 and MMP-9 are associated with aggressive cell behaviors, such as migration and invasion [47]. Moreover, emerging evidence indicates that MMPs are related to the induction of EMT. Furthermore, inhibition of the Wnt/β-catenin pathway by XAV939 impaired the promotive effects of HORMAD1 overexpression on EMT and cell motility, whereas activation of the Wnt/β-catenin pathway by CHIR99021 reversed the suppressive effects of HORMAD1 KO. Therefore, the HORMAD1-induced malignant phenotypes of lung cancer cells correlate with activation of the Wnt/β-catenin pathway.

In addition, we revealed the signaling mechanism by which HORMAD1 regulates the Wnt/β-catenin in lung cancer cells. Our results demonstrated that HORMAD1 overexpression increased Ser473 phosphorylation of AKT and Ser9 phosphorylation of GSK-3β, which attenuated the degradation of β-catenin and promoted its nuclear translocation. Moreover, the GSK-3β inhibitor Licl partially restored the levels of p-GSK-3β (Ser9) and β-catenin, which were initially reduced by HORMAD1 KO. Furthermore, treatment of HORMAD1-overexpressing cells with the specific AKT inhibitor MK-2206 partially restored the levels of p-GSK-3β (Ser9) and p-β-catenin (Ser33/37/Thr41), accompanied by partial reversal of the HORMAD1 overexpression-induced changes in the expression level and transcriptional activity of β-catenin. These results indicate that HORMAD1 activates the Wnt/β-catenin pathway partially through AKT/GSK-3β signaling. However, whether additional signaling is involved in HORMAD1-mediated regulation of the Wnt/β-catenin pathway should be investigated further.

In conclusion, we revealed that HORMAD1 functions as an oncogenic marker to promote lung cancer growth and metastasis. Additionally, this study provides the first evidence that HORMAD1 activates the Wnt/β-catenin pathway to promote EMT through modulation of AKT/GSK-3β signaling. This study provides new insights into the protumorigenic role of HORMAD1 and the regulatory mechanism by which it regulates the Wnt/β-catenin pathway in lung cancer progression and indicates that HORMAD1 may be a potential therapeutic target for lung cancer.

## Materials and methods

### Bioinformatic analysis

HORMAD1 mRNA expression data from pan-lung cancer, lung adenocarcinoma, lung squamous cell carcinoma and normal lung tissues were downloaded from the TCGA Data Portal and log2 transformed. The mRNA-seq data were analyzed as previously described [48]. In brief, R package was used to extract the expression values of HORMAD1 for statistical analyses and the Qlucore Omics Explorer (QOE 3.1) bioinformatics software was used to generate a heatmap of these expression data.

### Tissue microarray assay and immunohistochemistry analysis

Human lung adenocarcinoma tissue microarrays containing 91 lung adenocarcinoma tissues and the corresponding normal lung tissues (HLugA180Su04) were purchased from Outdo Biotech Company (Shanghai, China). Clinicopathological information including age, gender, tumor grade, etc., can be obtained from the manufacturer’s website (http://www.superchip.com.cn). Immunohistochemical staining of the lung adenocarcinoma tissue microarray was performed using a Leica BOND-MAX automated stainer (Leica Biosystems, Wetzlar, Germany). Briefly, the paraffin sections were deparaffinized, treated with antigen retrieval buffer, and heated. The sections were then stained first with the indicated antibodies and then with a horseradish peroxidase (HRP)-conjugated goat-anti-mouse or rabbit secondary antibody (Jackson ImmunoResearch, West Grove, PA, USA). Signals were detected using the chromogenic substrate DAB. Slides were counterstained with hematoxylin and were then mounted. The total score of positive staining was calculated as staining intensity score multiplied by the percentage of stained positively cells. The staining intensity was scored as follows: 0 points for negative staining; 1 point for weak intensity; 2 points for moderate intensity; 3 points for strong intensity. The percentage of stained positively cells were scored as: 1 point for 25% positive cells; 2 points for 26–50% positive cells; 3 points for 51–75% positive cells; 4 points for more than 75% positive cells. A total score ≥4 was considered as significant overexpression.

### Cell culture and transfection

Lung cancer cell lines and a normal human lung fibroblast cell line were obtained from the American Type Culture Collection (ATCC, Manassas, VA, USA) and cultured in Dulbecco’s modified Eagle medium (DMEM, Thermo Fisher Scientifific, Waltham, MA, USA) supplemented with 10% fetal bovine serum (FBS, Gibco, Carlsbad, CA, USA) and 1% penicillin and streptomycin. All cell lines were confirmed to be free of mycoplasma infection and were authenticated by short tandem repeat profiling. Transient cell transfection was performed with the indicated plasmids using Lipofectamine 2000 (Invitrogen, Carlsbad, CA, USA) according to the manufacturer’s instructions. For the Small-interfering RNA (siRNA) transfection, cells were transfected twice at an interval of 24 h with siRNA duplexes (100 nm) using Lipofectamine RNAiMAX (Invitrogen, Carlsbad, CA, USA) according to the manufacturer’s instructions. siRNA duplexes targeting β-catenin (5′-GCAAGGUGCAUCCUGAAAUTT-3′) and ZEB-1 (5′-GGAUGAAGAGGAACCCAAATT-3′) and HORMAD1 (1# 5′-CCAUGAGUGCACUGGUAUUTT-3′, 2# 5′-GCAUUCUCCUCAUUCGCAATT-3′) and the nontargeting control siRNA (5′-UUCAAUAAAUUCUUGAGGUUU-3′) were purchased from GenePharma (Shanghai, China).

### Antibodies and chemicals

Anti-β-catenin (sc-7963), anti-E-cadherin (sc-8426), and anti-Vimentin (sc-6260) antibodies were purchased from Santa Cruz (Dallas, TX, USA). Anti-GSK-3β (#12456), anti-p-GSK-3β (#9322), anti-AKT (#4691), anti-p-AKT (#4060), and anti-p-β-catenin (#9561) antibodies were purchased from Cell Signaling Technology (California, USA). Anti-HORMAD1 (67091-1-Ig), anti-ZEB-1 (21544-1-AP), anti-LaminB1 (12987-1-AP), and anti-N-cadherin (22018-1-AP) antibodies were purchased from Proteintech (Wuhan, China). Anti-α-tubulin (A01410-100) antibody was purchased from GenScript (Nanjing, China). The Wnt activator (CHIR99021), Wnt inhibitor (XAV939) and AKT inhibitor (MK-2206) were purchased from Selleckchem Chemicals (Houston, TX, USA).

### Immunofluorescent staining

Cells were plated on coverslips and fixed with 4% paraformaldehyde for 10 min. After permeabilization with 0.5% Triton X-100 for 5 min, cells were incubated with the indicated primary antibodies overnight at 4 °C. Cells were subsequently incubated with Alexa Fluor 488 or Alexa Fluor 594 (Life Technologies, Carlsbad, CA, USA) labeled secondary antibodies for 2 h at room temperature. Cells were then stained with DAPI and visualized with a fluorescence microscope (Leica Camera AG, Wetzlar, Germany).

### Western blotting

Cells were harvested and lysed with RIPA buffer containing protease and phosphatase inhibitors for 10 min on ice. Proteins in the supernatants were denatured with protein loading buffer and were then separated by SDS–PAGE. Proteins were transferred to PVDF membranes, and the membranes were then blocked in 5% milk and incubated with the indicated primary antibodies for 1 h at room temperature. The membranes were subsequently incubated with the appropriate HRP-conjugated secondary antibodies for 2 h, and signals were visualized using an ECL detection system.

### Generation of HORMAD1-overexpressing cell lines

S-FLAG-streptavidin binding protein (SFB) triple-tagged HORMAD1 expression plasmid and empty vector were separately transfected into cells. After transfection for 24 h, cells were cultured in medium containing 2 μg/ml puromycin for 7 days. Cell lines that stably expressed SFB-tagged HORMAD1 and the corresponding control cells were selected and confirmed by western blotting analysis.

### Generation of HORMAD1 knockout cell lines

The guide RNA sequences designed to target the human HORMAD1 were 5′-TCTTCACTAACACCAAAGAC-3′ (KO1) and 5′-TCCTGTATCACGTATTTGAG-3′ (KO2). The guide RNA sequences were cloned into the PX459 V2.0 vector (a gift from Feng Zhang, Addgene 62988) following the standard protocols. Cells were cultured in medium containing 2 μg/ml puromycin for 48 h after transfection with PX459 V2.0 containing the guide RNA constructs. Individual clones resistant to puromycin were selected and confirmed by western blotting analysis after 2 weeks.

### Cell proliferation and colony formation assays

For the proliferation assay, 1 × 10^4^ cells were seeded into each well of 96-well plates. Cell proliferation was measured by CCK8 reagent (Beyotime, Shanghai, China) at the indicated time points following the manufacturer’s instructions. For the colony formation assay, 1 × 10^3^ cells were plated in six-well plates. After cultured for 10 days, cells were fixed with 4% paraformaldehyde for 10 min and were then stained with 0.1% crystal violet. Colonies were counted using ImageJ software.

### Wound healing assay

Cells were seeded onto six-well plates and grown for 24 h until a confluent monolayer formed. The cell monolayers were scratched with a 10 μl pipette tip. After washing with PBS, the cells were cultured with serum-free medium and allowed to migrate for 48 h. Images were acquired at 0 h and 48 h and were then analyzed with ImageJ software.

### Transwell migration and Matrigel invasion assays

Cells were plated in the upper chambers of Transwell inserts (Corning, NY, USA) and cultured in serum-free medium. The lower chambers were filled with medium containing 10% FBS. For the Matrigel invasion assay, the upper chambers were precoated with polymerized Matrigel before the cells were plated. After culture for 24 h, the cells adhering to the upper surface of the chamber membrane were removed by wiping, and the cells adhering to the bottom surface of the chamber membrane were fixed with 4% paraformaldehyde for 10 min and stained with 0.1% crystal violet. Images were acquired using a fluorescence microscope for further analysis.

### Cytoplasmic and nuclear protein extraction

Cytoplasmic and nuclear protein extracts were separated by using Nuclear and Cytoplasmic Extraction Reagents (Sangon Biotech, Shanghai, China) according to the manufacturer’s instructions. The extracts were then analyzed by western blotting.

### Quantitative real-time PCR

Total RNA from cell lines was extracted using TRIzol reagent (Invitrogen, Carlsbad, CA, USA) according to the manufacturer’s instructions. cDNA was synthesized using a PrimeScriptTM RT cDNA Synthesis Kit (Takara Biomedical Technology, Beijing, China). A TB Green Premix Kit (Takara Biomedical Technology, Beijing, China) was used to perform quantitative real-time PCR amplification on the cDNA in an ABI 7900HT Real-Time PCR system (Applied Biosystems, Foster City, CA, USA). The relative mRNA levels of target genes were normalized to the level of GAPDH and calculated by the 2^-△△CT^ method.

### Dual luciferase reporter assay

A total of 2 × 10^4^ cells were seeded into each well of 24-well plates. Twenty-four hours later, the cells were cotransfected with the TOP-Flash or FOP-Flash reporter plasmid and the pRL-TK plasmid (Promega, Madison, WI, USA) as the negative control. After 48 h of incubation, luciferase activity was measured using a dual-luciferase reporter assay system (Promega, Madison, WI, USA) according to the manufacturer’s instructions.

### Animal study

A total of 5 × 10^6^ cells were injected subcutaneously into the armpit of male BALB/c nude mice (4-weeks-old, *n* = 5 per group) for the xenograft model to evaluate tumor growth. The tumor volume was calculated using the following equation: *V* = (width^2^ × length)/2. After 5 weeks, the mice were sacrificed, and the tumors were removed for weighing and photographing. In addition, mouse models of lung and liver metastasis were established to evaluate tumor metastasis. For the lung metastasis model, 3 × 10^6^ cells were injected into the tail vein of BALB/c nude mice (6-week-old, *n* = 5 per group). The liver metastasis model was established as described previously [26]. Briefly, 3 × 10^6^ cells were injected into the subcapsular region of male BALB/c nude mice (6-week-old, *n* = 5 per group). The mice were sacrificed after 6 weeks. The lungs and livers were removed for nodules counting and H&E staining to confirm tumor metastasis. For xenograft tumor formation and metastasis model, the mice were randomly divided into two or three groups. All mouse experiments were approved by the Experimental Animal Ethics Committee of Zhejiang University.

### Statistical analysis

All data in bar and line graphs are presented as mean ± SEM of at least three independent experiments. Data were analyzed using one-way ANOVA or the two-tailed unpaired Student’s *t*-test using GraphPad Prism version 7.0 for comparisons between multiple or two groups, respectively. Statistical significance is denoted as follows: **p < 0.01, ***p < 0.001; ns, not significant. Kaplan-Meier analysis was used to establish overall survival curves. The log-rank test was used to compare the overall survival of lung adenocarcinoma patients between the high and low HORMAD1 expression groups. *P* < 0.05 was considered as statistically significant.

## Supplementary information

Supplementary Figures

Western blotting original data

## Data Availability

The datasets used and/or analyzed during the current study are available from the corresponding author on reasonable request.
