# Supplementary material for: The cancer/testis antigen HORMAD1 mediates epithelial–mesenchymal transition to promote tumor growth and metastasis by activating the Wnt/β-catenin signaling pathway in lung cancer
Source: Cell Death Discov. 2022 Mar 28;8:136. doi: 10.1038/s41420-022-00946-1 (PMC8960869; doi:10.1038/s41420-022-00946-1)
Supplement: Supplementary file 1 — Supplementary Figures [file 41420_2022_946_MOESM1_ESM.pdf]

Supplementary Figures

Figure S1

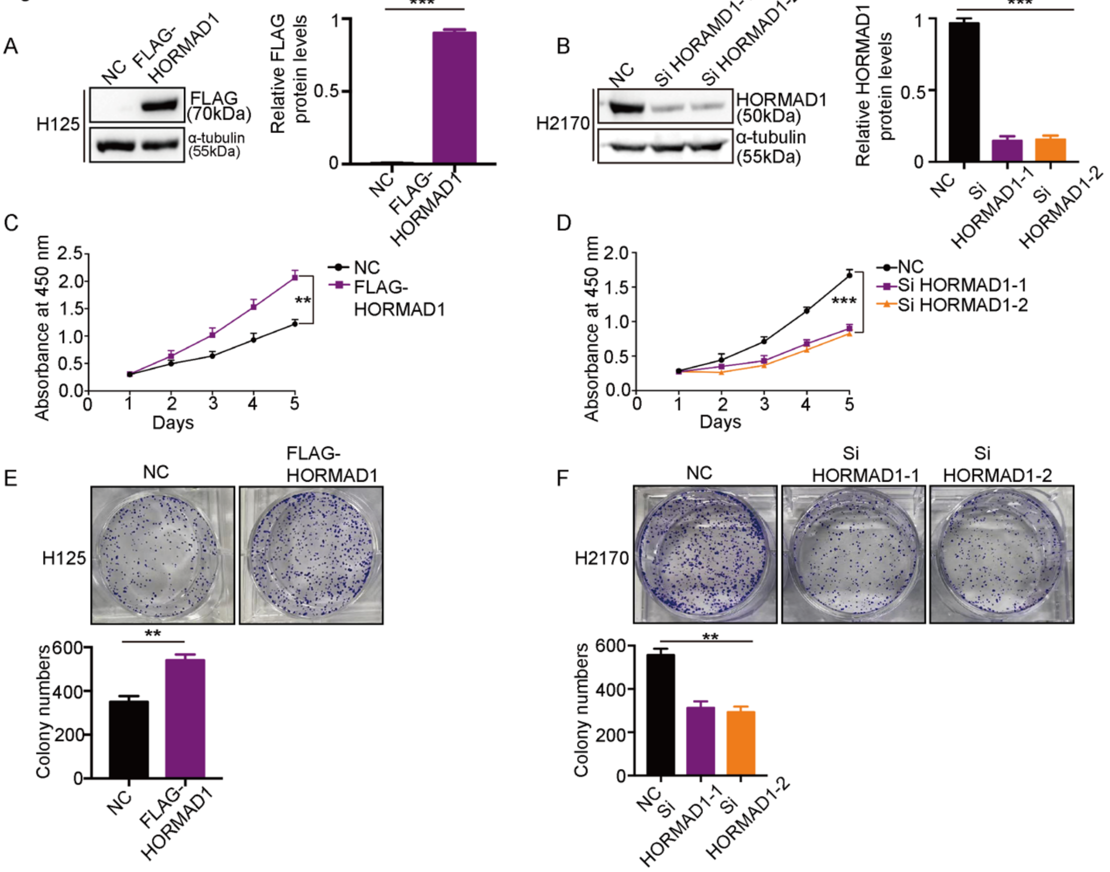

Figure S1. HORMAD1 promotes the proliferation of lung cancer cells *in vitro*.

A. Western blotting and quantitative analyses of HORMAD1 expression in H125 cells with or without HORMAD1 expression. α-tubulin was used as the loading control. **B.** Western blotting and quantitative analyses of HORMAD1 expression in HORMAD1 negative control (NC) and knockdown H2170 cells. α-tubulin was used as the loading control. **C-D.** The proliferation of H125 cells with or without HORMAD1 expression (**C**) and NC and HORMAD1 knockdown H2170 cells (**D**) was measured by CCK-8 assays at the indicated time points. **E-F.** The colony-forming ability of H125 cells with or without HORMAD1 expression (**E**) and NC and HORMAD1 knockdown H2170 cells (**F**) was measured by colony formation assays. All histograms representing

indicated the results of three independent experiments. Mean ± SEM from three independent experiments are shown. **, p＜0.01; ***, p＜0.001.

Figure S2

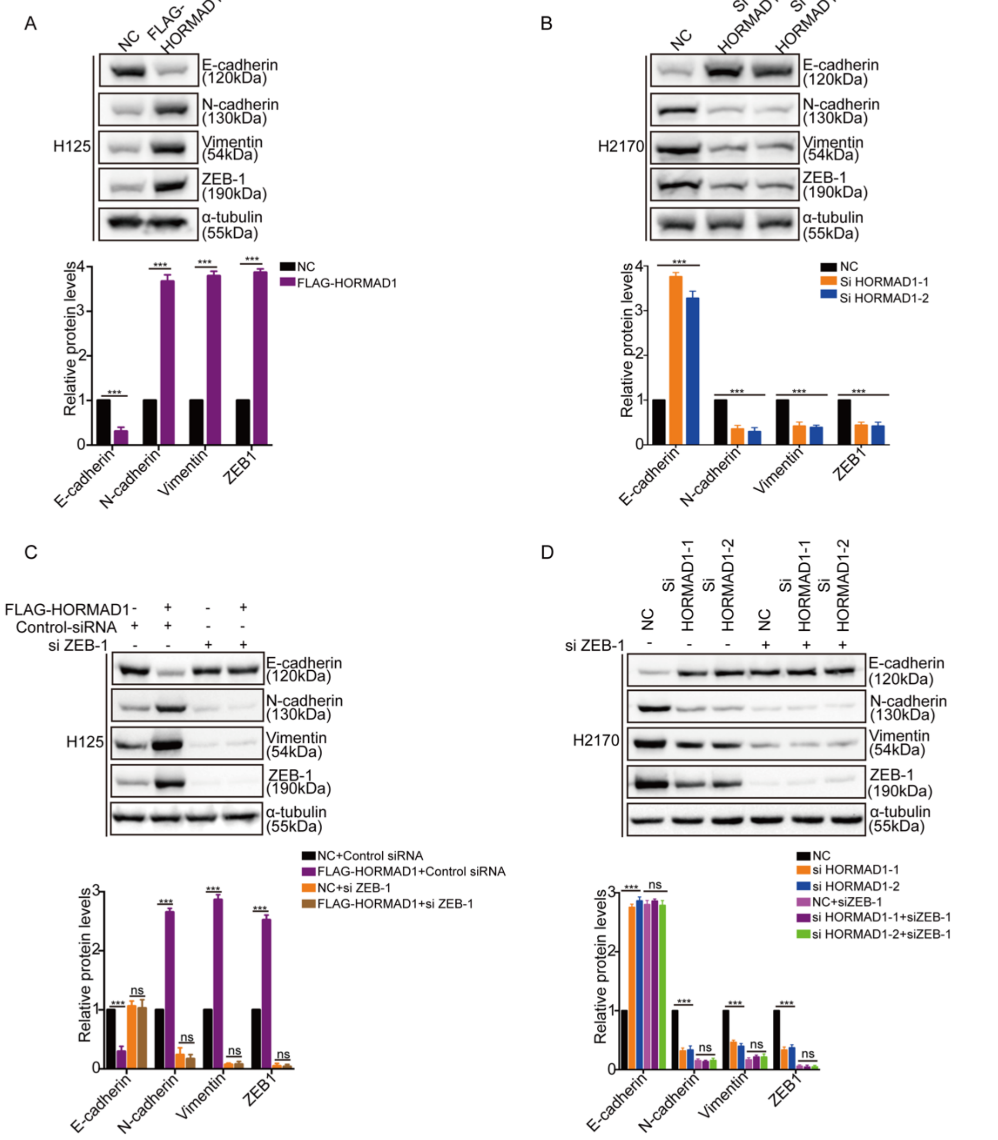

Figure S2. HORMAD1 promotes epithelial-mesenchymal transition (EMT) in lung cancer cells.

A-B. The protein levels of E-cadherin, N-cadherin, Vimentin, and ZEB-1 in H125 cells with or without HORMAD1 expression (A) and in NC and HORMAD1 knockdown

H2170 cells (B) were analyzed by western blotting. **C-D.** The protein levels of E-cadherin, N-cadherin, Vimentin, and ZEB-1 in H125 cells with or without HORMAD1 expression (**C**) and in NC and HORMAD1 knockdown H2170 cells (**D**) were analyzed by western blotting after transfection with ZEB-1 siRNA. All histograms representing indicated the results of three independent experiments. Mean ± SEM from three independent experiments are shown. ***, p＜0.001; ns, not significant.

Figure S3

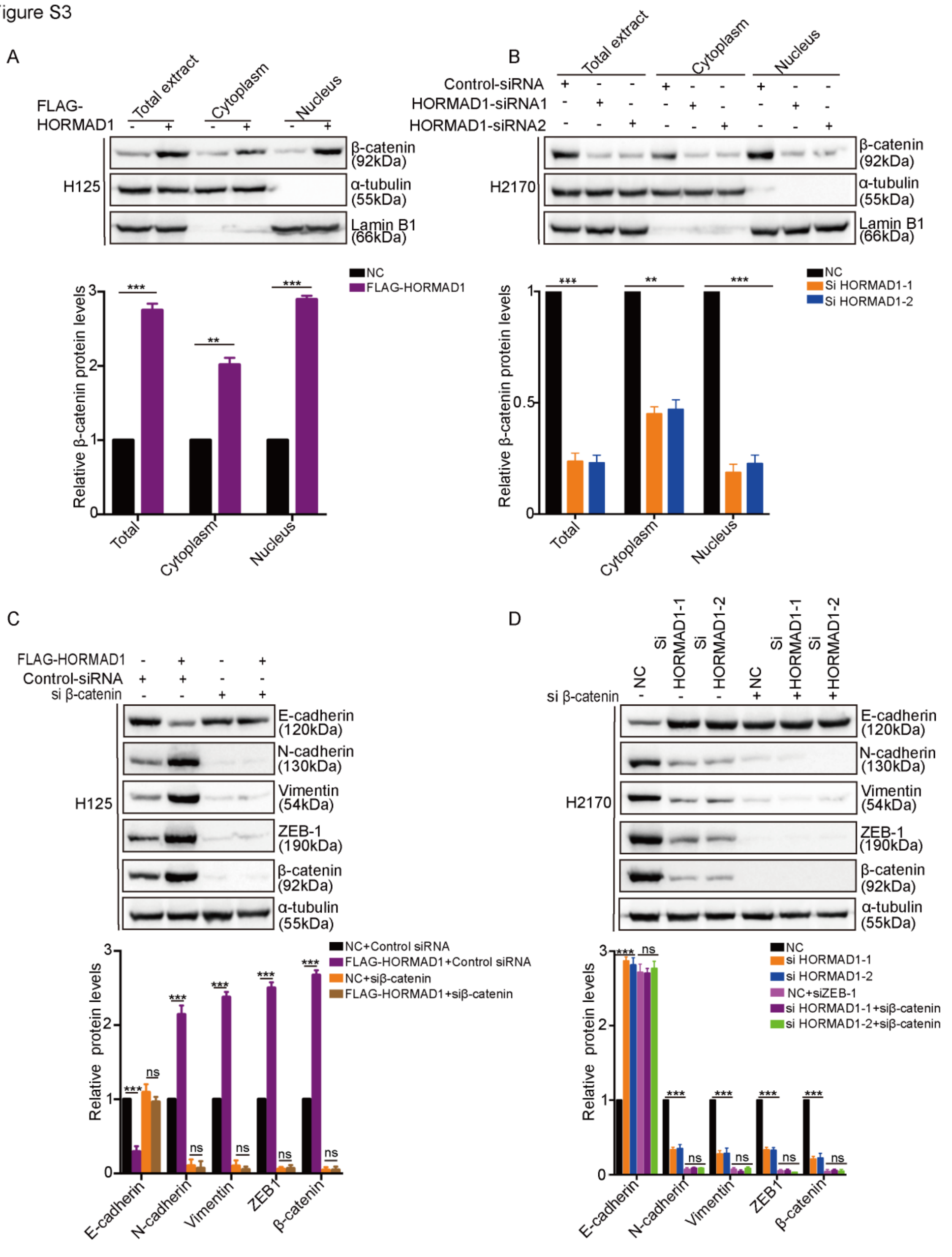

Figure S3. HORMAD1 activates the Wnt/β-catenin pathway in lung cancer cells.

A-B. Western blotting and quantitative analyses of total, cytosolic, and nuclear β-catenin in H125 cells with or without HORMAD1 expression (**A**) and in NC and HORMAD1 knockdown H2170 cells (**B**). α-tubulin and Lamin B1 were used as the loading controls for the cytoplasmic and nuclear fractions, respectively. **C-D.** Western blotting and quantitative analyses of E-cadherin, N-cadherin, Vimentin, ZEB-1, and β-catenin protein levels in H125 cells with or without HORMAD1 expression (**C**) and in NC and HORMAD1 knockdown H2170 cells (**D**) after transfection with β-catenin siRNA. All histograms representing indicated the results of three independent experiments. Mean ± SEM from three independent experiments are shown. **, $p < 0.01$; ***, $p < 0.001$; ns, not significant.
