# Supplementary material for: The cancer/testis antigen HORMAD1 mediates epithelial–mesenchymal transition to promote tumor growth and metastasis by activating the Wnt/β-catenin signaling pathway in lung cancer
Source: Cell Death Discov. 2022 Mar 28;8:136. doi: 10.1038/s41420-022-00946-1 (PMC8960869; doi:10.1038/s41420-022-00946-1)
Supplement: Supplementary file 2 — Western blotting original data [file 41420_2022_946_MOESM2_ESM.pdf]

Original western blots in Figure 1C

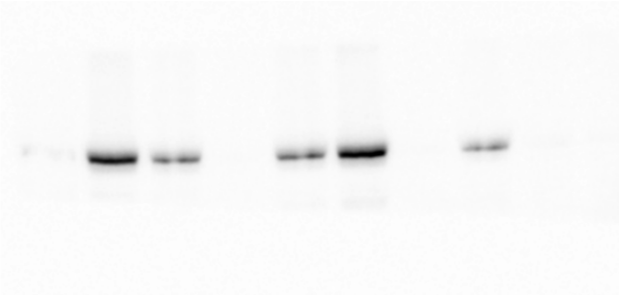

HORMAD1

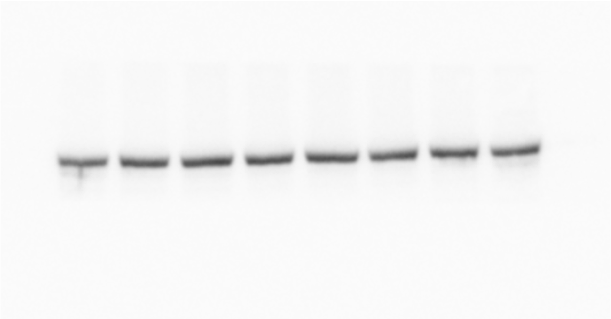

α -tubulin

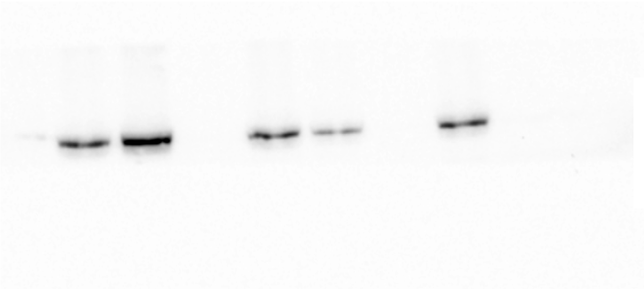

HORMAD1

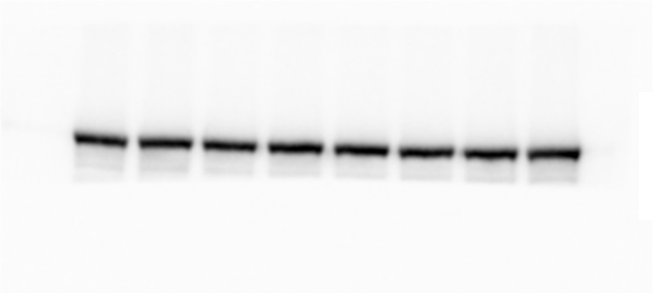

α -tubulin

Original western blots in Figure 2A

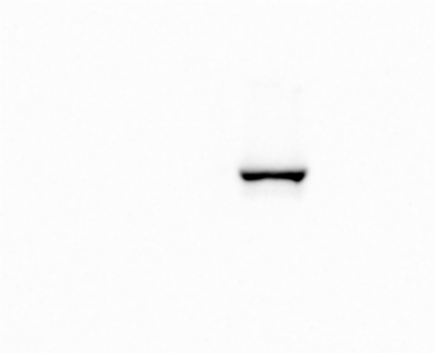

FLAG

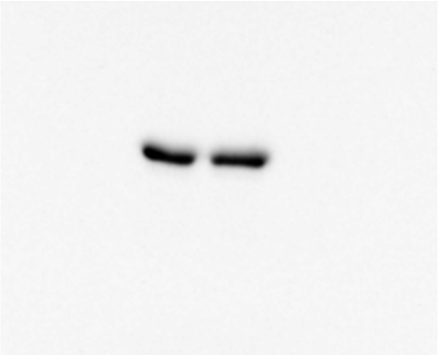

α -tubulin

Original western blots in Figure 2B

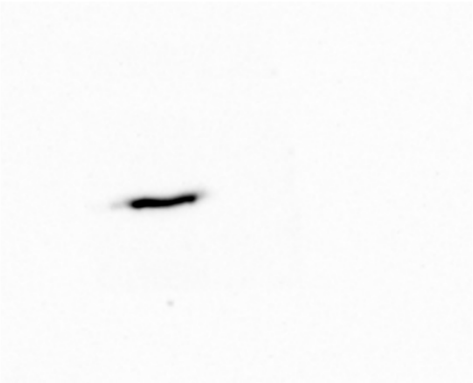

HORMAD1

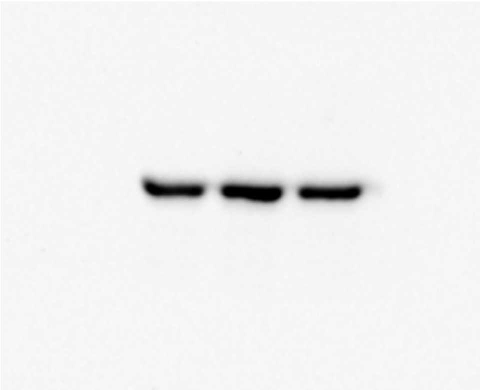

α -tubulin

Original western blots in Figure 4C

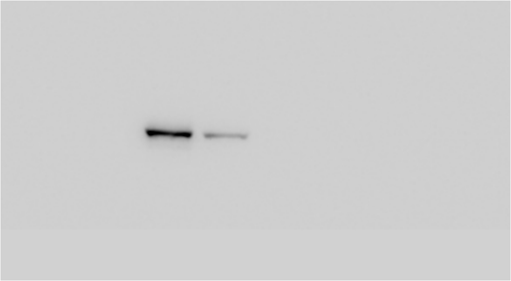

E-cadherin

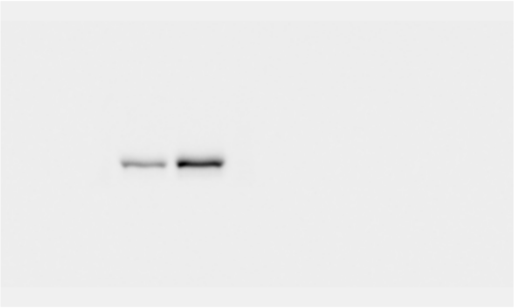

N-cadherin

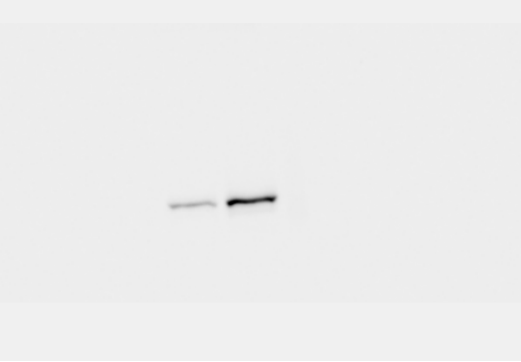

Vimentin

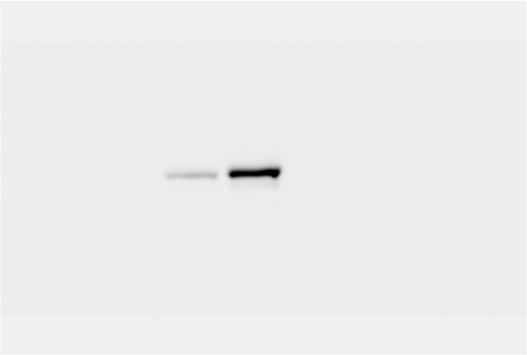

ZEB-1

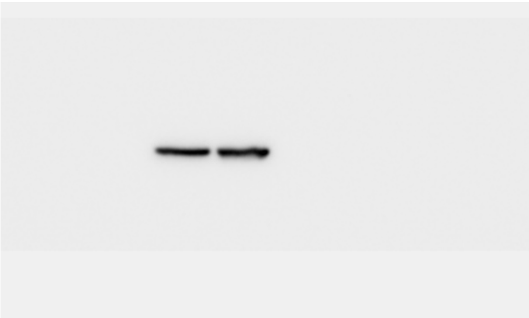

a-tubulin

Original western blots in Figure 4D

E-cadherin

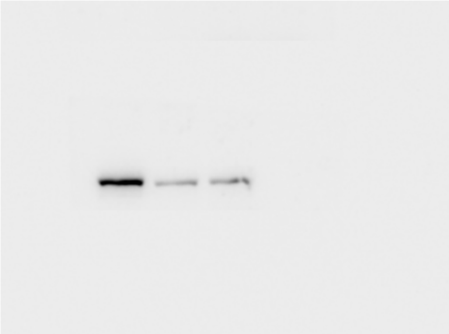

N-cadherin

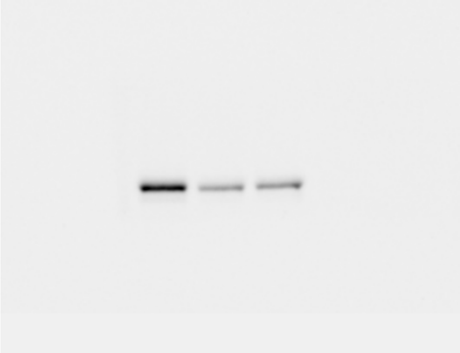

Vimentin

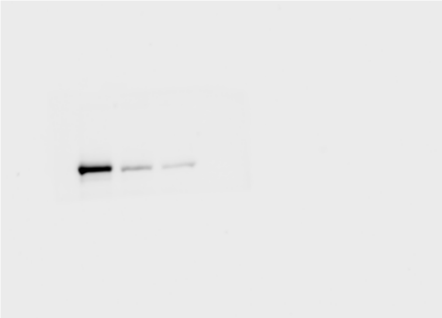

ZEB-1

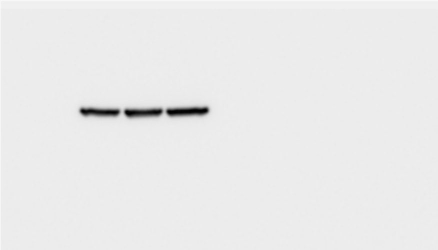

a-tubulin

Original western blots in Figure 4G

E-cadherin

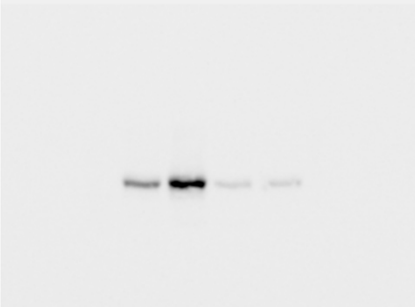

N-cadherin

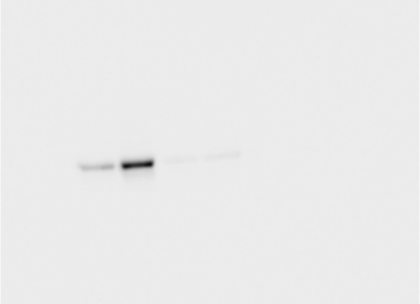

Vimentin

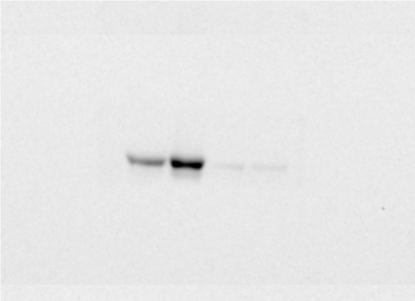

ZEB-1

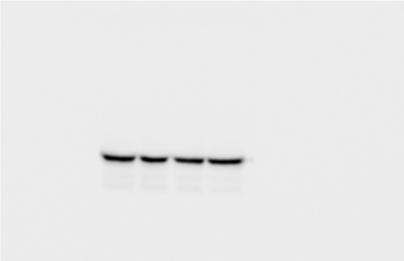

a-tubulin

Original western blots in Figure 4H

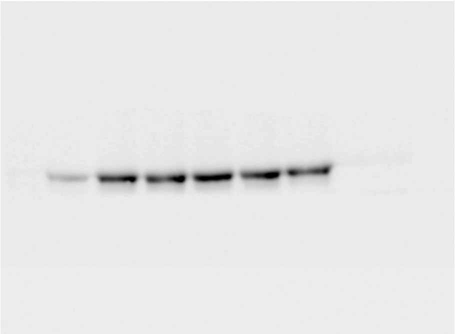

E-cadherin

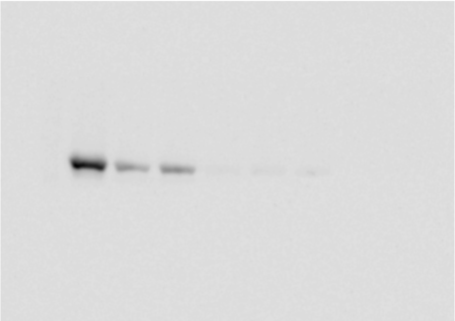

N-cadherin

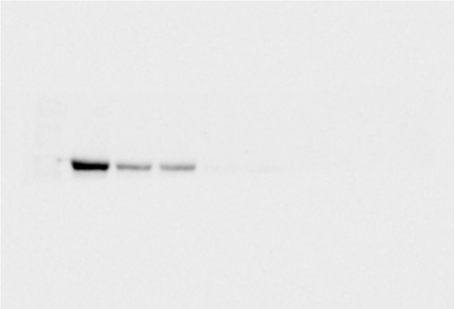

Vimentin

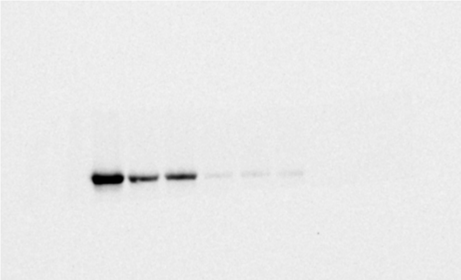

ZEB-1

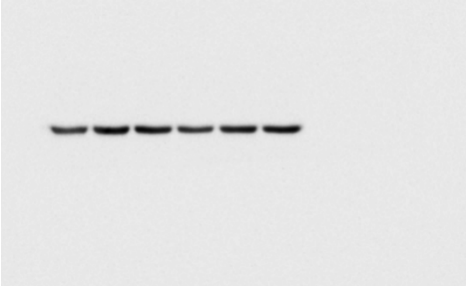

a-tubulin

Original western blots in Figure 5A

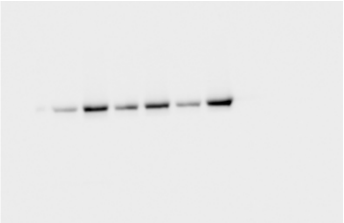

β -catenin

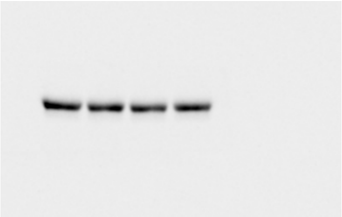

a-tubulin

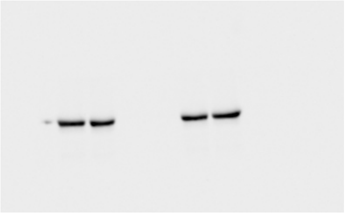

LaminB1

Original western blots in Figure 5B

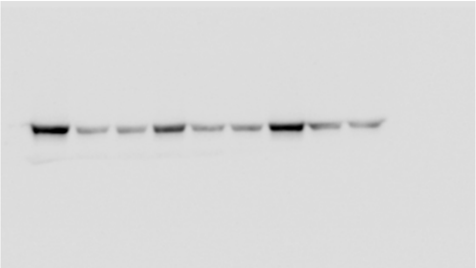

β -catenin

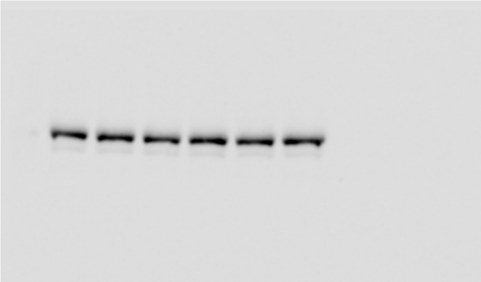

α -tubulin

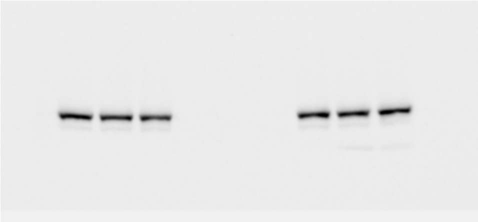

LaminB1

Original western blots in Figure 5I

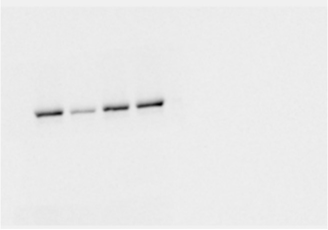

E-cadherin

N-cadherin

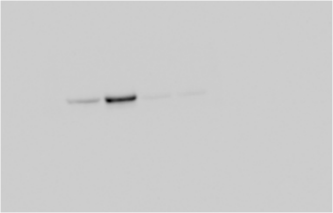

Vimentin

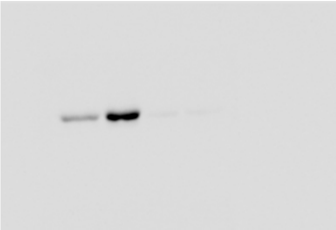

ZEB-1

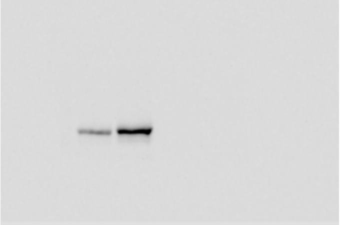

β -catenin

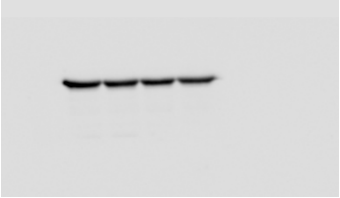

a-tubulin

Original western blots in Figure 5J

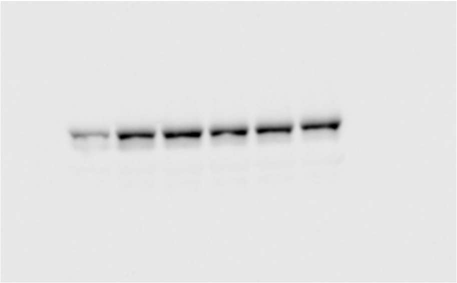

E-cadherin

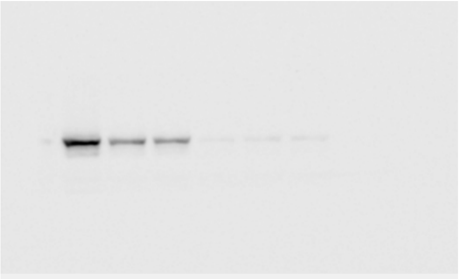

N-cadherin

Vimentin

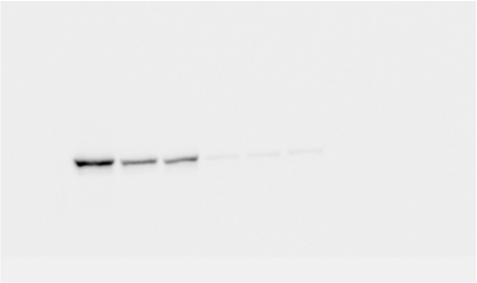

ZEB-1

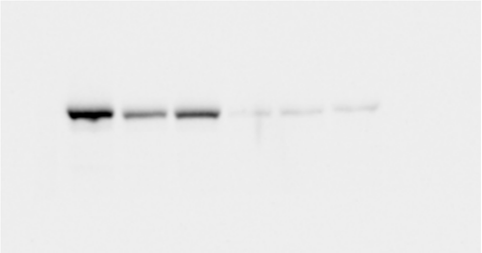

β -catenin

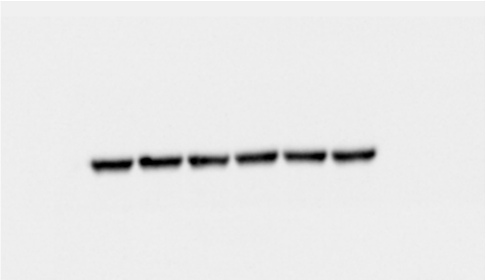

a-tubulin

Original western blots in Figure 6A

β-catenin

p-β-catenin

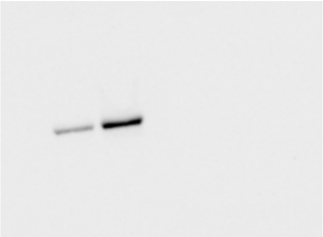

p-GSK3β

GSK3β

p-AKT

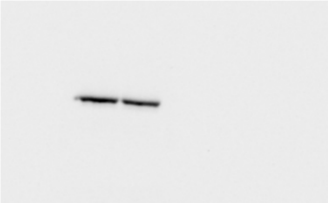

AKT

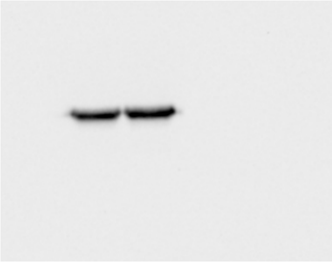

a-tubulin

Original western blots in Figure 6B

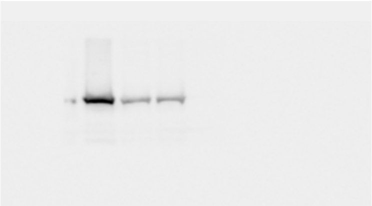

β-catenin

p-β-catenin

p-GSK3β

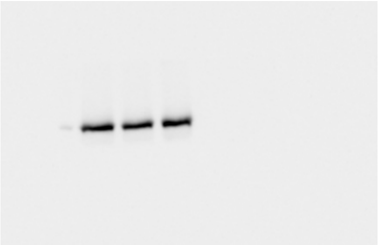

GSK3β

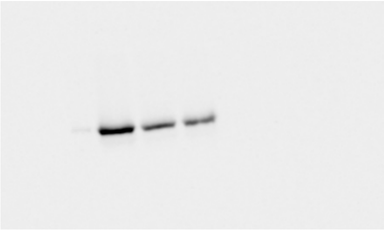

p-AKT

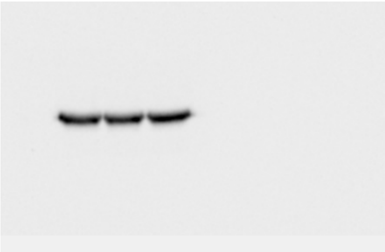

AKT

a-tubulin

Original western blots in Figure 6C

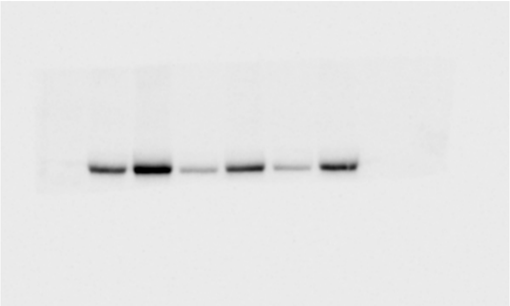

p-GSK3β

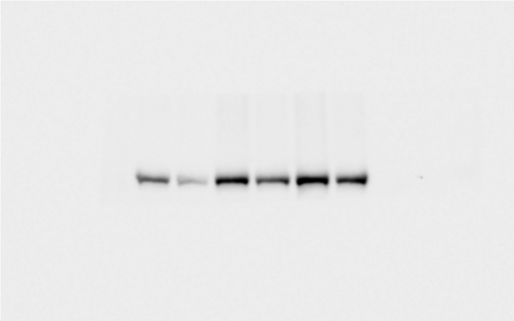

p-β-catenin

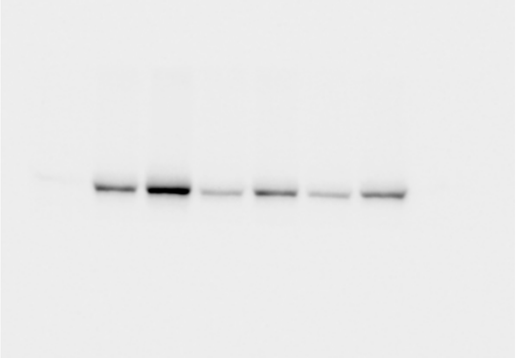

β-catenin

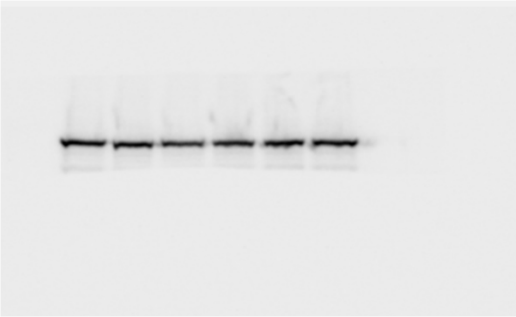

GSK3β

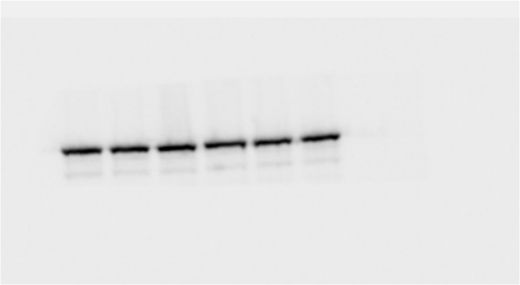

a-tubulin

Original western blots in Figure 6D

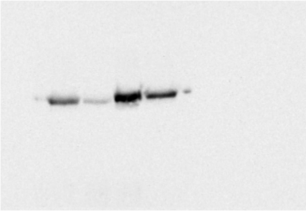

p-AKT

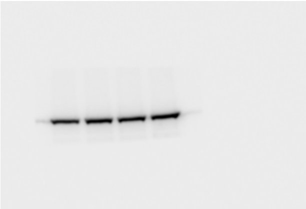

AKT

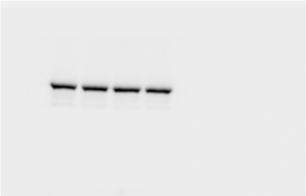

GSK-3 β

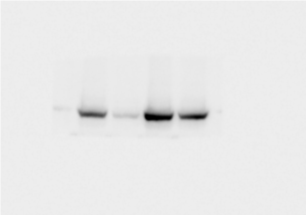

p-GSK-3 β

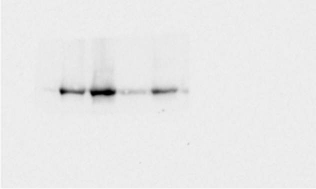

p- β -catenin

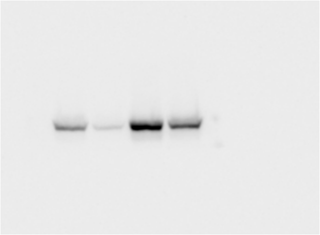

β -catenin

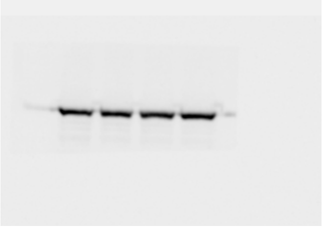

a-tubulin

Original western blots in Figure 7A

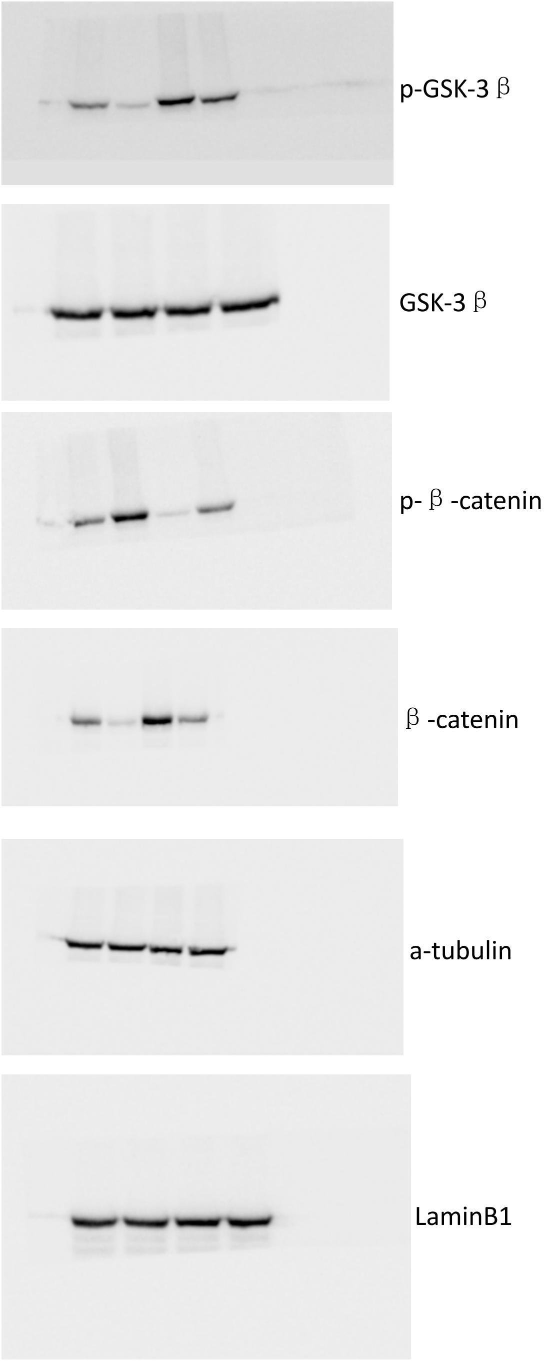

Original western blots in Figure 7B

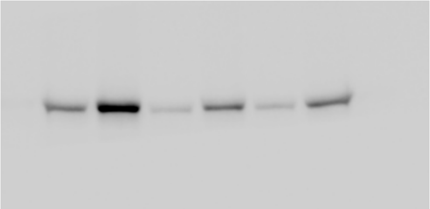

p-GSK-3 β

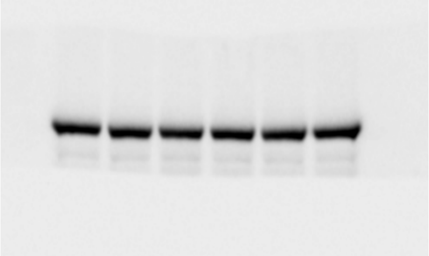

GSK-3 β

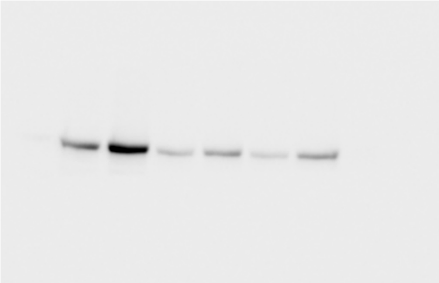

β -catenin

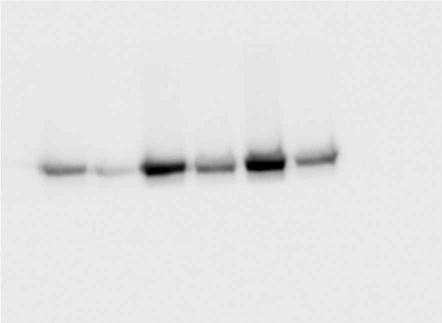

p- β -catenin

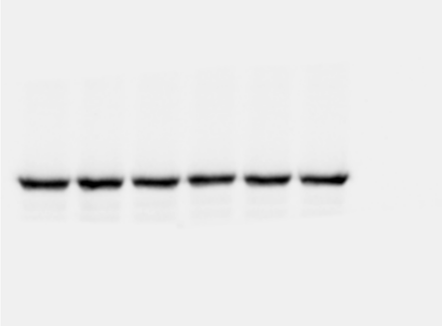

a-tubulin

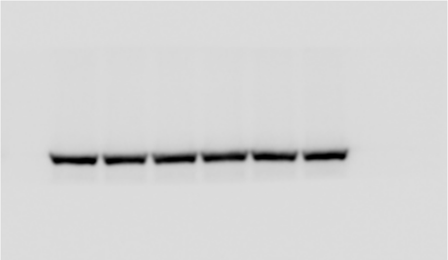

LaminB1

Original western blots in Figure 7E

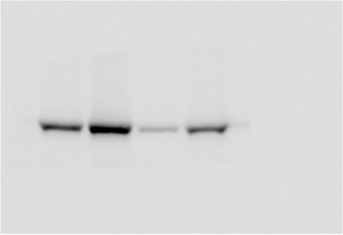

E-cadherin

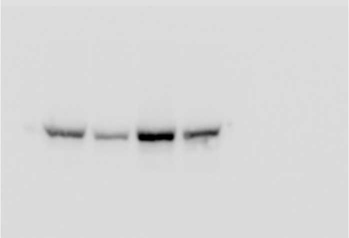

N-cadherin

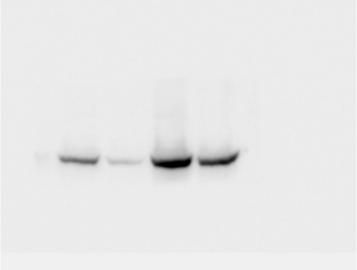

Vimentin

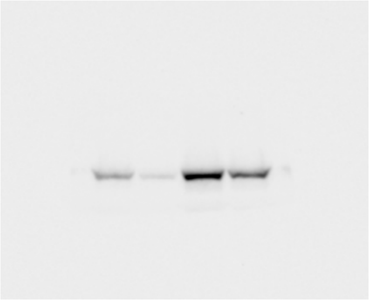

ZEB-1

a-tubulin

Original western blots in Figure 7F

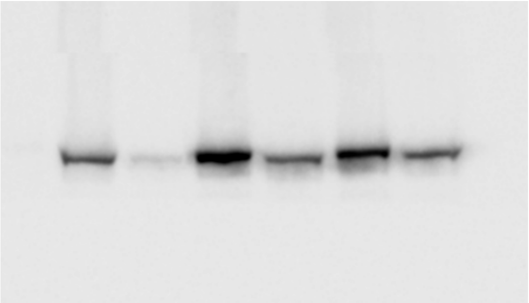

E-cadherin

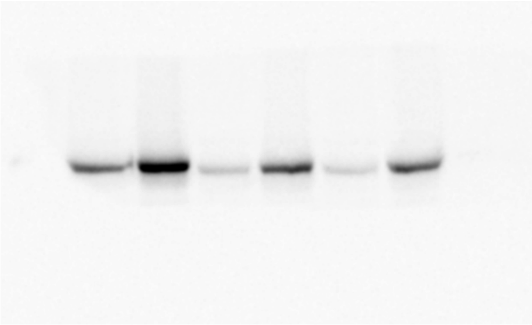

N-cadherin

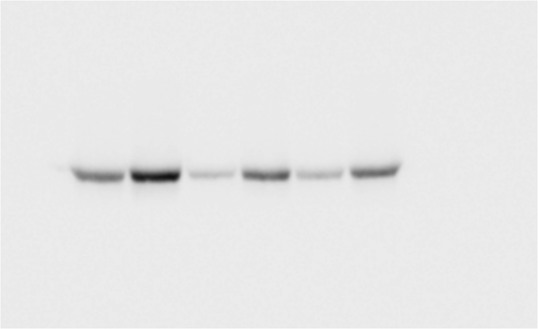

Vimentin

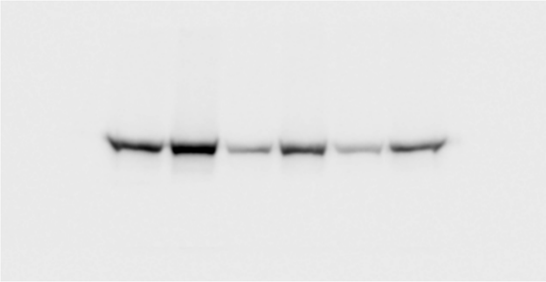

ZEB-1

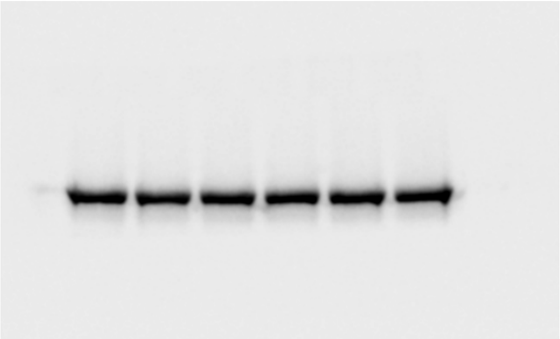

a-tubulin

Original western blots in Figure S1A

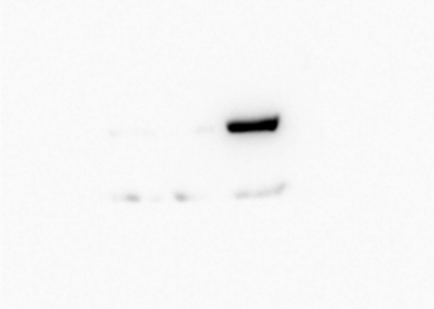

FLAG

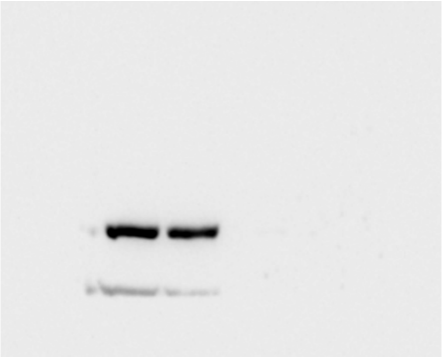

a-tubulin

Original western blots in Figure S1B

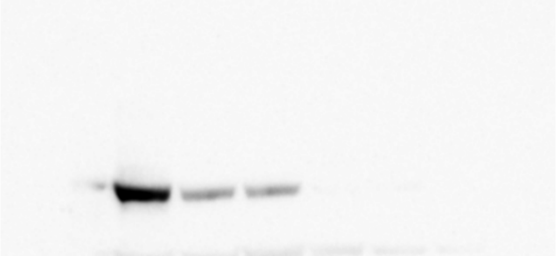

HORMAD1

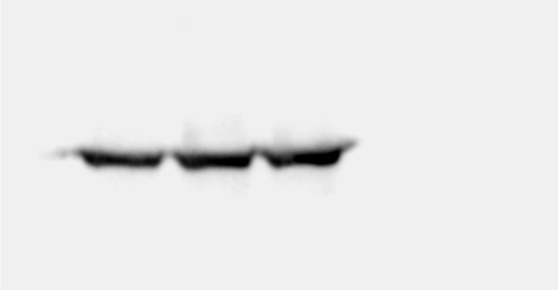

a-tubulin

Original western blots in Figure S2A

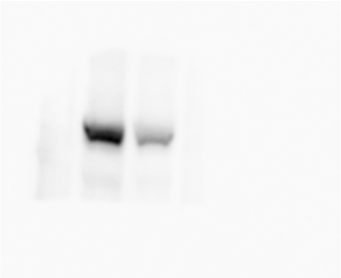

E-cadherin

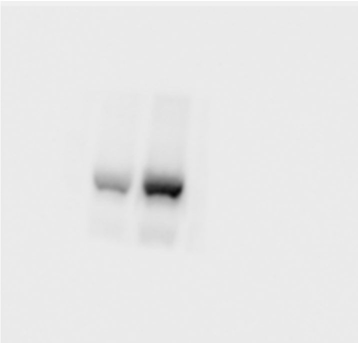

N-cadherin

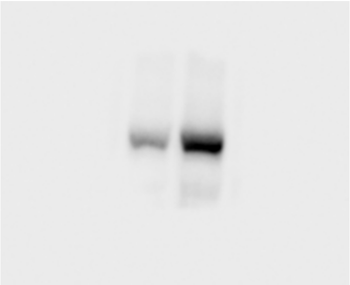

Vimentin

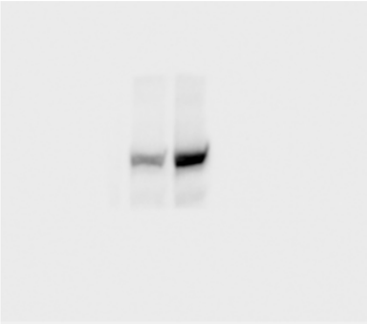

ZEB-1

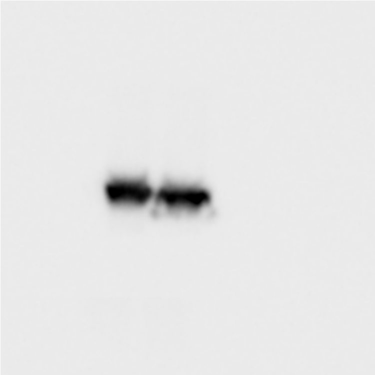

a-tubulin

Original western blots in Figure S2B

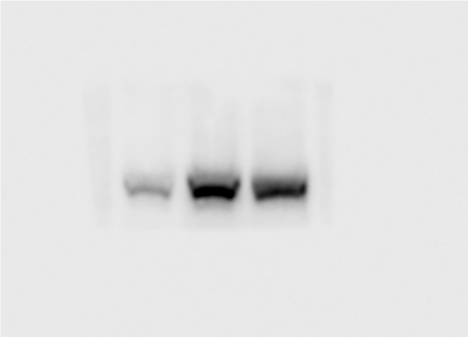

E-cadherin

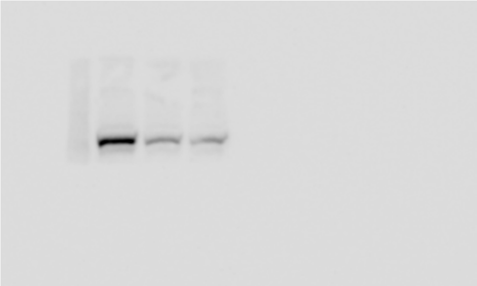

N-cadherin

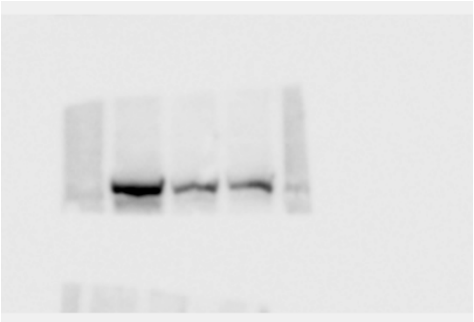

Vimentin

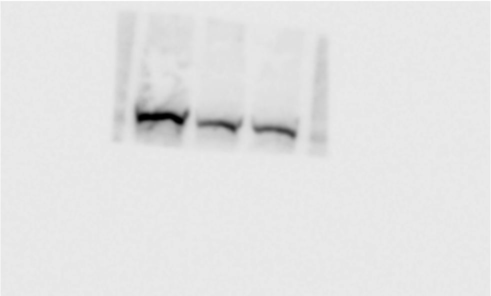

ZEB-1

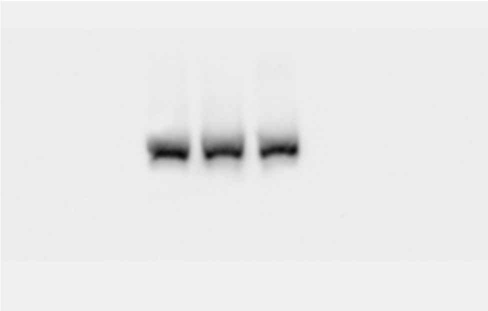

a-tubulin

Original western blots in Figure S2C

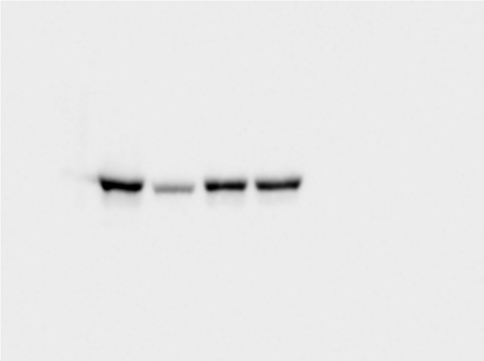

E-cadherin

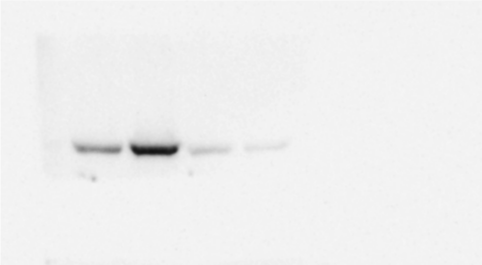

N-cadherin

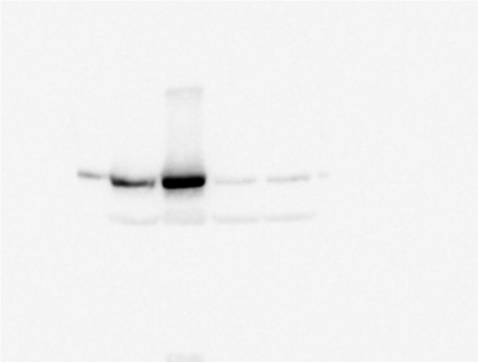

Vimentin

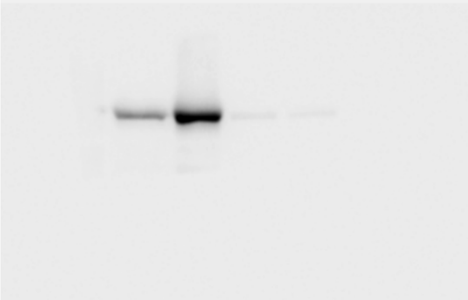

ZEB-1

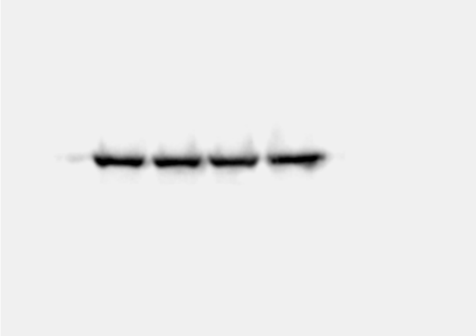

a-tubulin

Original western blots in Figure S2D

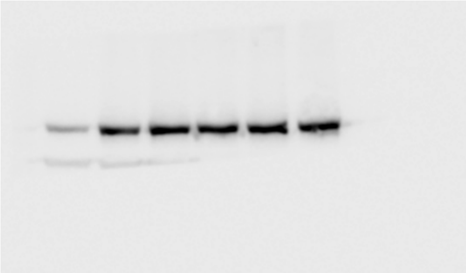

E-cadherin

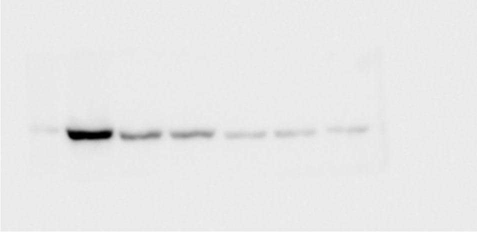

N-cadherin

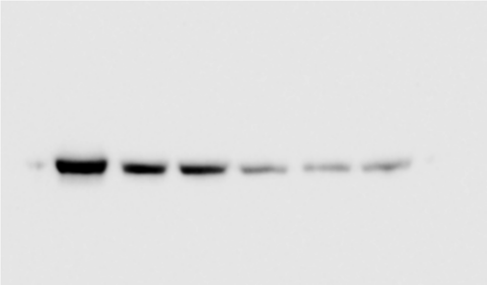

Vimentin

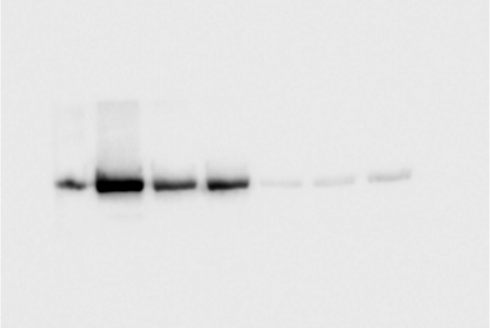

ZEB-1

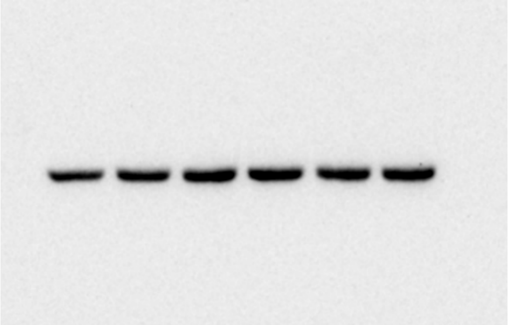

a-tubulin

Original western blots in Figure S3A

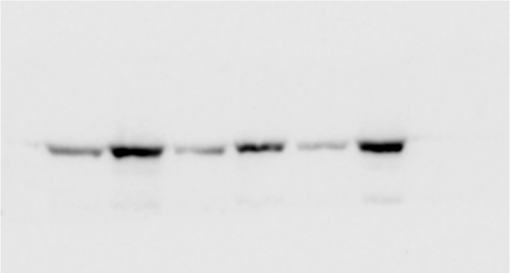

β -catenin

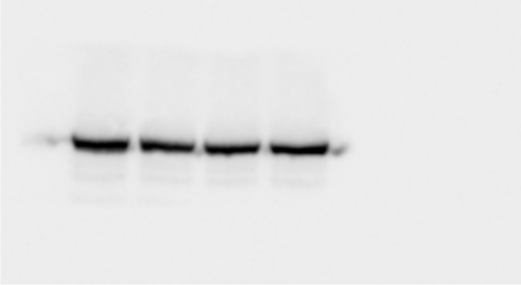

a-tubulin

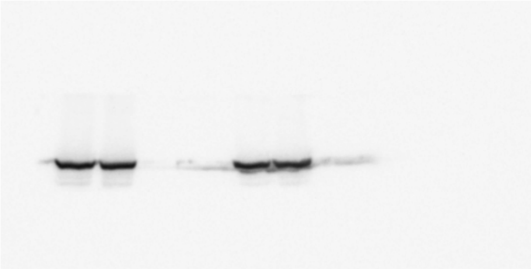

LaminB1

Original western blots in Figure S3B

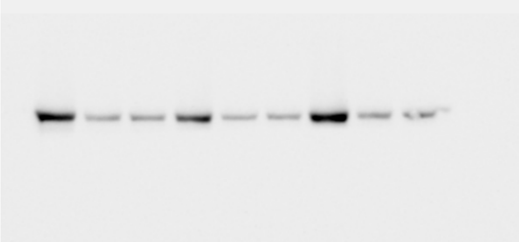

β -catenin

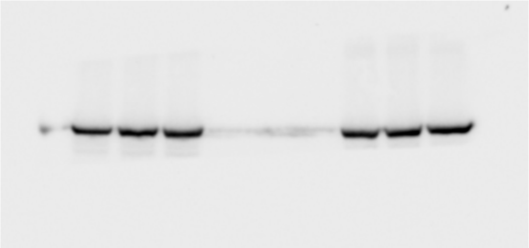

LaminB1

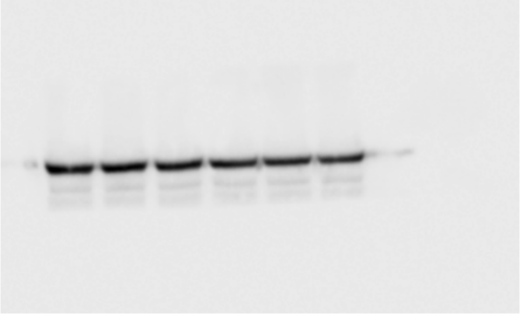

a-tubulin

Original western blots in Figure S3C

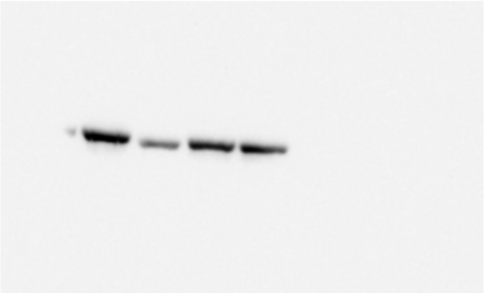

E-cadherin

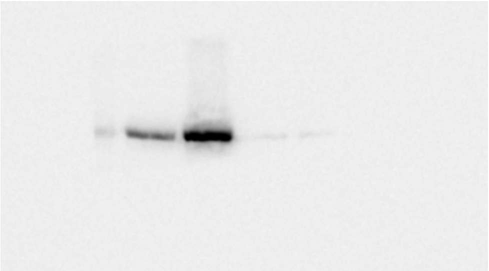

N-cadherin

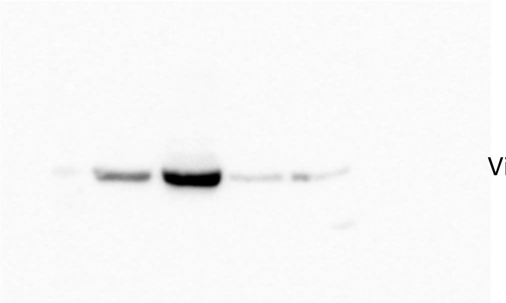

Vimentin

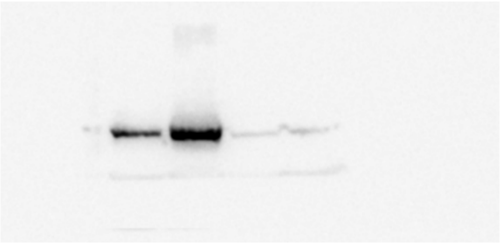

ZEB-1

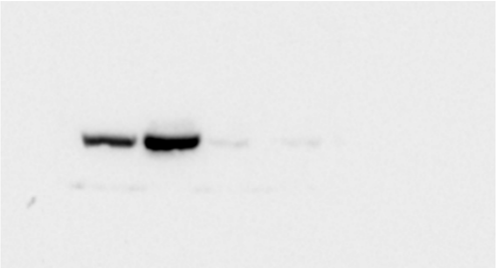

β -catenin

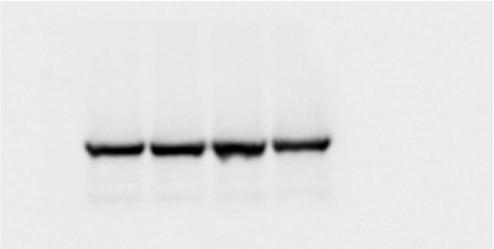

a-tubulin

Original western blots in Figure S3D

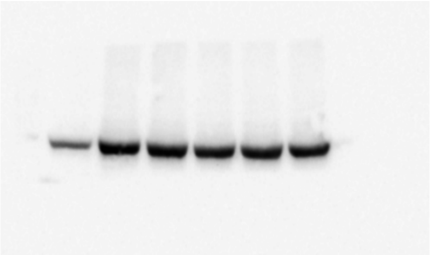

E-cadherin

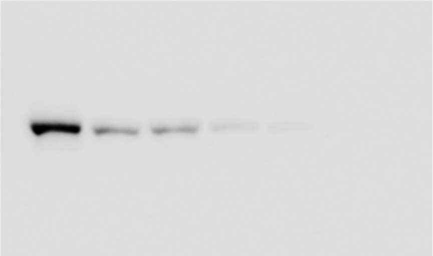

N-cadherin

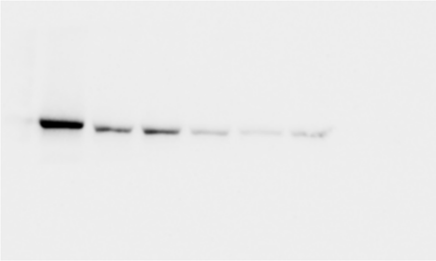

Vimentin

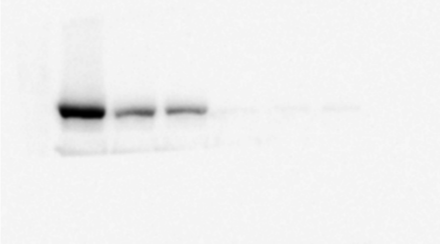

ZEB-1

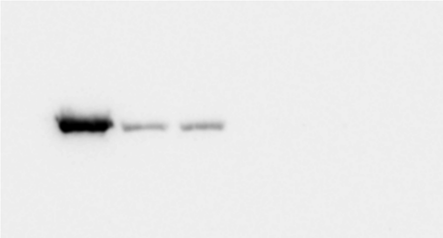

β -catenin

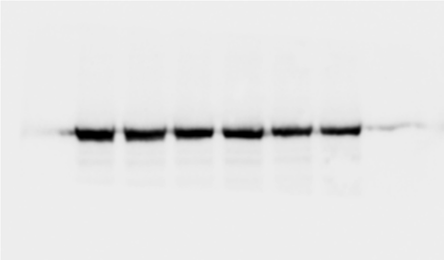

a-tubulin
